# CRISPR/Cas9 ribonucleoprotein-mediated knockout of *Gly m 4-L1* eliminates allergen accumulation in soybean

**DOI:** 10.3389/fpls.2026.1739979

**Published:** 2026-03-09

**Authors:** Shinya Chatani, Chikako Kuwabara, Miki Hibara, Sara Kazahaya, Ayaka Tange, Yuichiro Tamamoto, Toshiyuki Hirata, Yuma Fukutomi, Nobuyuki Maruyama, Tetsuya Yamada

**Affiliations:** 1 Graduate School of Agriculture, Hokkaido University, Hokkaido, Japan; 2 Graduate School of Agriculture, Kyoto University, Kyoto, Japan; 3 Field Science Center Northern Biosphere, Hokkaido University, Hokkaido, Japan; 4 NHO Sagamihara National Hospital, Kanagawa, Japan

**Keywords:** allergen, gene editing, Gly m 4, glycine max, hypoallergenic, PR-10, seed

## Abstract

Gly m 4 is a major allergen in soy milk and cross-reacts with Bet v 1, the primary birch pollen allergen. To reduce Gly m 4 allergenicity, we performed site-directed mutagenesis of the *Gly m 4* gene in soybean through a DNA-free *in planta* bombardment method using ribonucleoproteins (iPB-RNP). Although a mutant line (*Gly m 4-2^del^*) was generated by targeting the *Gly m 4-2* gene, immunoblot analysis revealed that translation products from other homologs still accumulated in the seed tissues. Subsequent gene expression analysis identified *Gly m 4-L1*, one of eight *Gly m 4* homologs in the soybean genome, as the primary target for further mutagenesis. Ribonucleoprotein complexes loaded with either a single guide RNA (gRNA) or two distinct gRNAs targeting *Gly m 4-L1* were introduced into the shoot apical meristems. Sequencing analysis identified three mutant lines: an 8-bp deletion (*Gly m 4-L1^8-del^*), a 128-bp insertion (*Gly m 4-L1^128-ins^*), and a complete gene deletion (*Gly m 4-L1^null^*). Immunoblot analysis confirmed the absence of Gly m 4 protein accumulation in the seeds of these mutants. To evaluate immunoglobulin E (IgE) reactivity, protein extracts from *Gly m 4-2^del^*, *Gly m 4-L1^null^*, and wild-type plants were incubated with sera from patients positive for Gly m 4–specific IgE. Protein extracts from the *Gly m 4-L1^null^* line showed markedly reduced IgE binding compared with *Gly m 4-2^del^* and wild-type samples. These findings demonstrate that *Gly m 4-L1* is the key gene responsible for Gly m 4 allergen production in soybean seeds.

## 1 Introduction

Soybean (*Glycine max*) is one of the most economically important leguminous crops due to the high-quality proteins in its seeds, which have amino acid profiles comparable to those of beef and egg white (Liu, 2004). Soybean protein is an essential resource driving the growth of the global plant-based food market (Ahmad et al., 2022). However, its utilization as a protein source is frequently challenged by its allergenicity. Food allergies represent a significant global health concern, affecting approximately 4.3% of the world’s population (Feng et al., 2023). To mitigate the risks associated with accidental exposure, the Codex Alimentarius Commission has designated eight major food groups—milk, eggs, peanuts, tree nuts, crustacean shellfish, fish, soybeans, and wheat—as “priority allergens” that require mandatory labeling (Boyce et al., 2010). These eight items account for the vast majority of food allergies (Sampson, 2004). Among legume allergens, peanut allergy is the most prevalent, affecting approximately 2% of the general population in Western nations (Lieberman et al., 2021). In contrast, soybean allergy accounts for 0.2–0.4% of allergic individuals worldwide; however, like peanut allergy, it can induce anaphylaxis and remains a serious clinical concern (Nakagawara et al., 2013; Wiederstein et al., 2023).

Soybean allergy develops through two distinct sensitization pathways. Primary sensitization occurs after direct ingestion of soybean proteins (Moroz and Yang, 1980; Ogawa et al., 1993), including Kunitz trypsin inhibitor (KTI), subunits of 7S and 11S globulins, 2S albumin, and the oil body–associated protein Gly m Bd 30K (Moroz and Yang, 1980; Ogawa et al., 1991; Ogawa et al., 1993; Gu et al., 2001; Holzhauser et al., 2009; Maruyama et al., 2018). Secondary sensitization results from cross-reactivity with pollen allergens and often occurs alongside primary sensitization. Representative proteins in this group include profilin (Gly m 3) (Rihs et al., 1999; González et al., 2000). The presence of allergenic proteins in soybean seeds remains a major obstacle for individuals with soy allergies. Therefore, developing soybean cultivars that lack these allergens is crucial to providing safer dietary options. However, progress in genetically reducing soybean allergenicity has been limited to date.

Identifying allergen-deficient alleles within soybean genetic resources can facilitate the development of novel cultivars with broader consumer acceptance. Hypoallergenic soybean lines can be generated by exploiting spontaneous mutants that lack or show reduced levels of allergenic proteins. Several mutants deficient in 7S globulin subunits have been reported (Kitamura et al., 1984; Takahashi et al., 1994; Hajika et al., 1996). Additionally, soybean germplasm includes variants with low KTI activity or KTI-deficient mutants (Jofuku et al., 1989; Krishnan, 2001). Variations in Gly m Bd 30K accumulation have also been observed in both cultivated and wild soybean accessions (Xu et al., 2007; Joseph et al., 2006; Bilyeu et al., 2009). Mutagenesis offers another effective strategy for generating allergen-deficient alleles. For example, gamma-ray irradiation has induced the absence of the α-subunit of 7S globulin and Gly m Bd 28K proteins (Samoto et al., 1997), while X-ray irradiation has generated mutant populations with reduced KTI activity (Ha et al., 2014).

The development of hypoallergenic soybean plants can also be advanced through biotechnological approaches. Expression of the *Gly m Bd 30K* gene can be suppressed by co-suppression or RNA interference (RNAi), reducing accumulation of the target protein in transgenic seeds (Herman et al., 2003; Liu et al., 2013). Similarly, 7S globulin subunit proteins in soybean seeds have been markedly reduced using RNAi or artificial microRNA systems (Nishizawa et al., 2010; Yamada et al., 2014). Site-directed mutagenesis has emerged as another effective strategy for generating allergen-deficient alleles. Mutant *Gly m Bd 30K* alleles have been generated using the clustered regularly interspaced short palindromic repeats (CRISPR)/CRISPR-associated protein 9 (Cas9) system delivered through *Agrobacterium*-mediated transformation or particle bombardment (Sugano et al., 2020; Adachi et al., 2021). Moreover, Sugano et al. (2020) generated mutant alleles of the *Gly m Bd 28K* gene simultaneously with *Gly m Bd 30K* through site-directed mutagenesis. More recently, a DNA-free *in planta* bombardment method using ribonucleoproteins (iPB-RNPs) was employed to generate Gly m Bd 30K–deficient mutants in four soybean genotypes (Kuwabara et al., 2024). Furthermore, CRISPR/Cas9-mediated *Agrobacterium* transformation has been used to develop mutant alleles at KTI loci (*KTI1* and *KTI3*) and 7S globulin subunit genes (Wang et al., 2023; Ha and Kim, 2023; Song et al., 2024). The mutants generated in these studies successfully achieved a dramatic reduction in the accumulation of the target allergen protein.

Similar to Gly m 3, Gly m 4 is characterized as a cross-reactive allergen that induces secondary sensitization in pollen-sensitized individuals (Kleine-Tebbe et al., 2002; Fukutomi et al., 2012). Notably, it is regarded as the primary allergen responsible for soy milk-induced allergic reactions. Most patients who exhibit allergic reactions to dietary soybean powder possess specific IgE antibodies against Gly m 4, as well as against the major birch pollen allergen Bet v 1 (*Betula platyphylla*) (Kleine-Tebbe et al., 2002; Fukutomi et al., 2012). The three-dimensional structure of Gly m 4 closely resembles that of Bet v 1 (Berkner et al., 2009), confirming the cross-reactivity between birch pollen sensitization and soy-based food allergy. Consequently, individuals allergic to birch pollen frequently react to Gly m 4 (Kleine-Tebbe et al., 2002; Mittag et al., 2004; Fukutomi et al., 2012), and Gly m 4 also exhibits cross-reactivity with other pollen allergens (Wiederstein et al., 2023). The *Gly m 4* gene was originally identified as starvation-associated message 22 (SAM22), which is induced in young soybean leaves by wounding, salicylic acid, fungal elicitors, and hydrogen peroxide (Crowell et al., 1992). Subsequent studies reclassified SAM22 as a pathogenesis-related 10 (PR-10) protein (Graham et al., 2003). The predicted PR-10 amino acid sequence was deposited in UniProtKB/Swiss-Prot under accession number P26987 (Crowell et al., 1992) and has since served as the reference for immunological and biochemical analyses of Gly m 4 (Mittag et al., 2004; Havenith et al., 2017; Zhou et al., 2022). Despite these insights, no genetic improvement using Gly m 4–deficient mutant alleles has been reported to date. In this study, site-directed mutagenesis was used to generate mutant alleles of the *Gly m 4* gene. The iPB-RNP system was employed because the soybean genotype used was recalcitrant to conventional *Agrobacterium*-mediated mutagenesis. Both frameshift mutations and a null allele of *Gly m 4* were successfully induced. Allergy testing with proteins fractionated from mutant seeds revealed a marked reduction in patient serum reactivity to Gly m 4. These findings demonstrate the potential of the iPB-RNP system as an effective strategy for generating hypoallergenic Gly m 4–deficient soybean plants.

## 2 Results

### 2.1 Selection of the *Gly m 4* gene and guide RNA design

**Figure 1:**
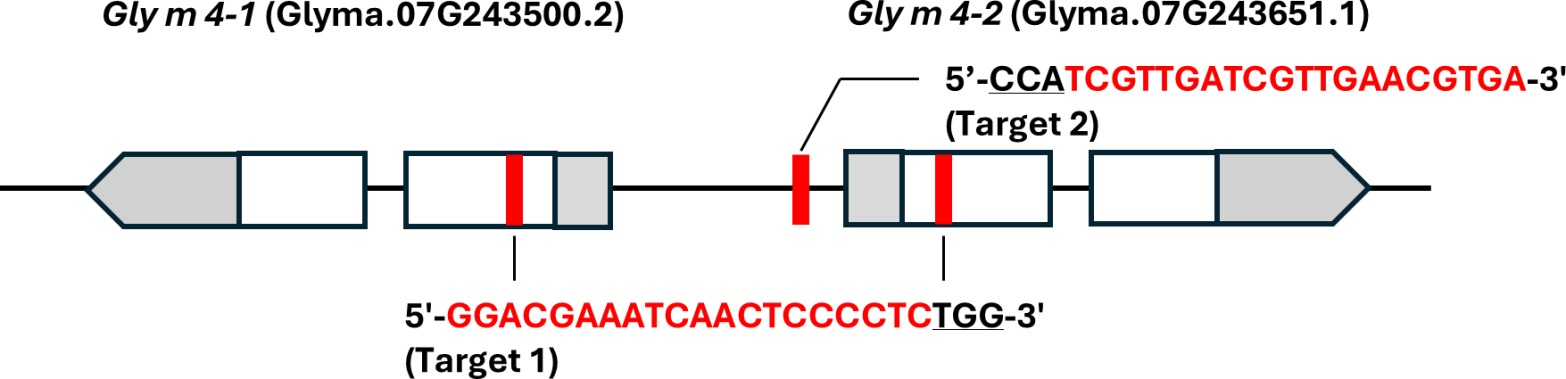
Schematic of site-directed mutagenesis of the *Gly m 4–1* and Gly *m 4–2* genes using the iPB-RNP system. Exons are shown as boxes and pentagons, with gray regions representing untranslated sequences; pentagons indicate transcriptional endpoints. Red vertical lines mark gRNA target sites. Sequences of the gRNA target regions are shown, with underlined nucleotides indicating the protospacer adjacent motif (PAM). Brackets below the sequences denote the names of the respective gRNA target sites.

The predicted Gly m 4 amino acid sequence was deposited in UniProtKB/Swiss-Prot under accession number P26987 (Crowell et al., 1992) and has since served as the reference for immunological and biochemical analyses of Gly m 4 (Mittag et al., 2004; Havenith et al., 2017; Zhou et al., 2022). A protein BLAST search against the Phytozome database (https://phytozome-next.jgi.doe.gov/) revealed that the amino acid sequences of *Glyma*.07G243500.2 and *Glyma*.07G243651.1 were identical to P26987. These two genes were therefore designated *Gly m 4–1* and *Gly m 4-2*, respectively. Two guide RNA (gRNA) target sites were selected: a common site within the first exons of *Gly m 4–1* and *Gly m 4-2* (Target 1), and an intergenic site positioned between the two genes (Target 2) (**Figure 1**). Site-directed mutagenesis of the *Gly m 4–1* and *Gly m 4–2* genes was performed using the iPB-RNP method. Plantlets regenerated from the bombarded embryonic axes were designated the E_0_ generation.

### 2.2 Selection and characterization of the *Gly m 4* mutant plant

**Figure 2:**
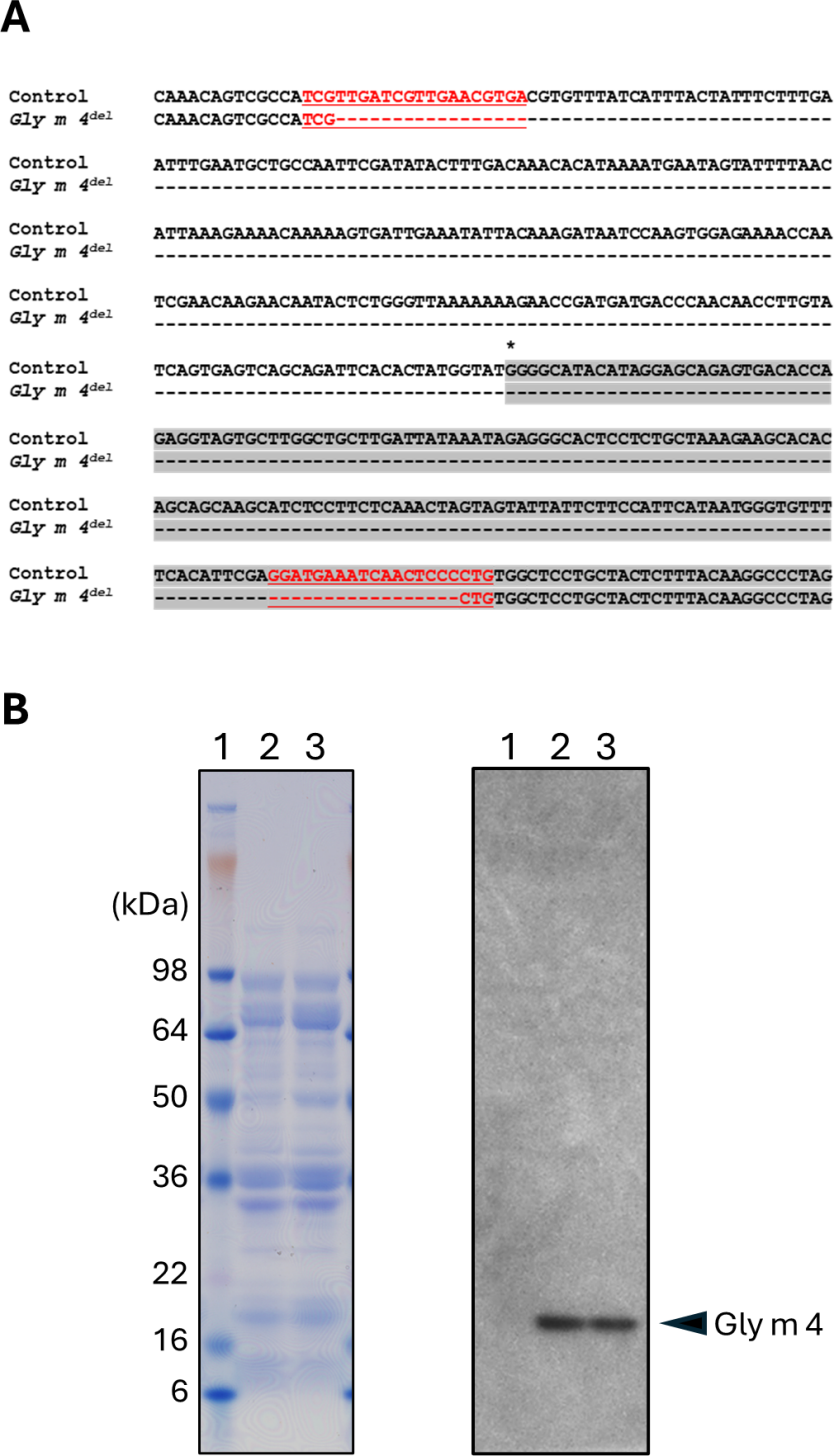
Nucleotide sequence of the *Gly m 4–2* locus in the *Gly m 4-2^del^* mutant and protein accumulation of Gly m 4 in control and *Gly m 4-2^del^* mutant plants. **(A)** Nucleotide sequence of the *Gly m* 4–2 locus and its flanking region in the *Gly m 4-2^del^* mutant. Red underlined nucleotides indicate the gRNA target site designed in this study. Gray-highlighted nucleotides represent exon regions. An asterisk denotes the transcription start site. **(B)** SDS-PAGE and immunoblot analysis of total seed proteins. Lanes 1, protein molecular weight markers; lane 2, control; lane 3, *Gly m 4-2^del^* mutant. The immunoblot (right panel) was probed with a polyclonal anti–Gly m 4 antibody.

Mutations in the target regions of E_0_ plants were analyzed by PCR and *in vitro* digestion using CRISPR/Cas9 ribonucleoprotein (RNP) assays on bulked genomic DNA extracted from multiple leaves per plant (Kuwabara et al., 2024). Among 59 seedlings screened, one E_1_ mutant was identified in which the region spanning from the Target1 site within *Gly m 4–2* to Target2 had been deleted (Kuwabara et al., 2024). A homozygous mutant allele was subsequently obtained in the E_2_ generation through self-pollination (**Supplementary Figure 1**). This mutation involved a 431-nucleotide deletion spanning the promoter region and the first exon of the *Gly m 4–2* gene (**Figure 2A**). The resulting mutant line was designated *Gly m 4-2^del^*. Immunoblotting using a polyclonal anti–Gly m 4 antibody was conducted to assess protein accumulation in seeds. Notably, strong antibody-reactive protein signals were observed in both *Gly m 4-2^del^* and control seeds (**Figure 2B**).

### 2.3 Selection of additional *Gly m 4* homologs for site-directed mutagenesis

**Figure 3:**
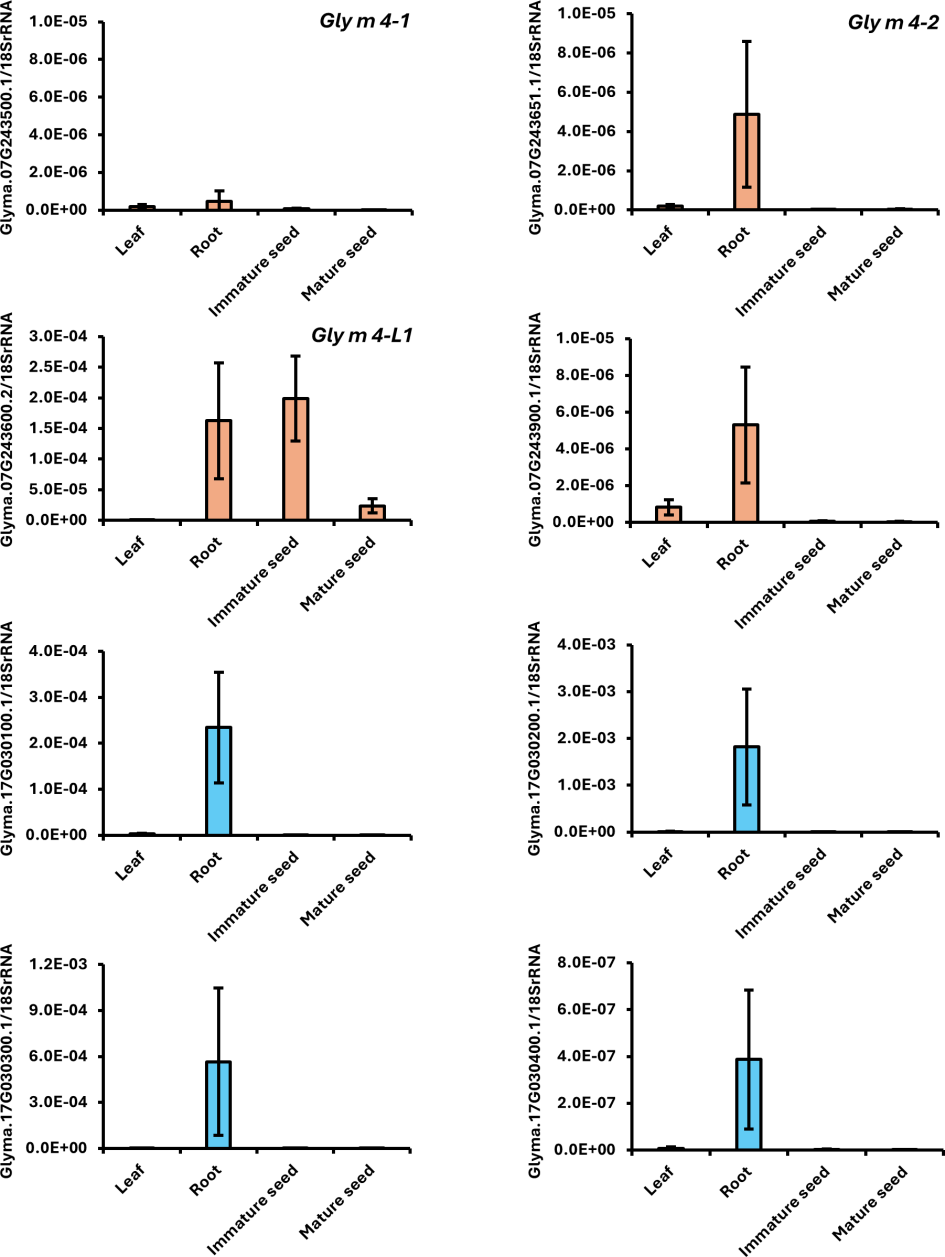
Expression of Gly m 4 and its homologs in Yukihomare measured by qRT-PCR. Gene identifiers correspond to *Glycine max* Wm82.a6.v1 annotations from Phytozome 13 (https://phytozome-next.jgi.doe.gov/). The gene targeted for site-directed mutagenesis (Glyma.07G243600.2) is designated *Gly m 4-L1*. Relative expression was normalized to 18S rRNA (XR_003264275). Data are means ± SE of three biological replicates. Graphs with aligned Y-axis scales are provided in **Supplementary Figure 4**.

**Figure 4:**
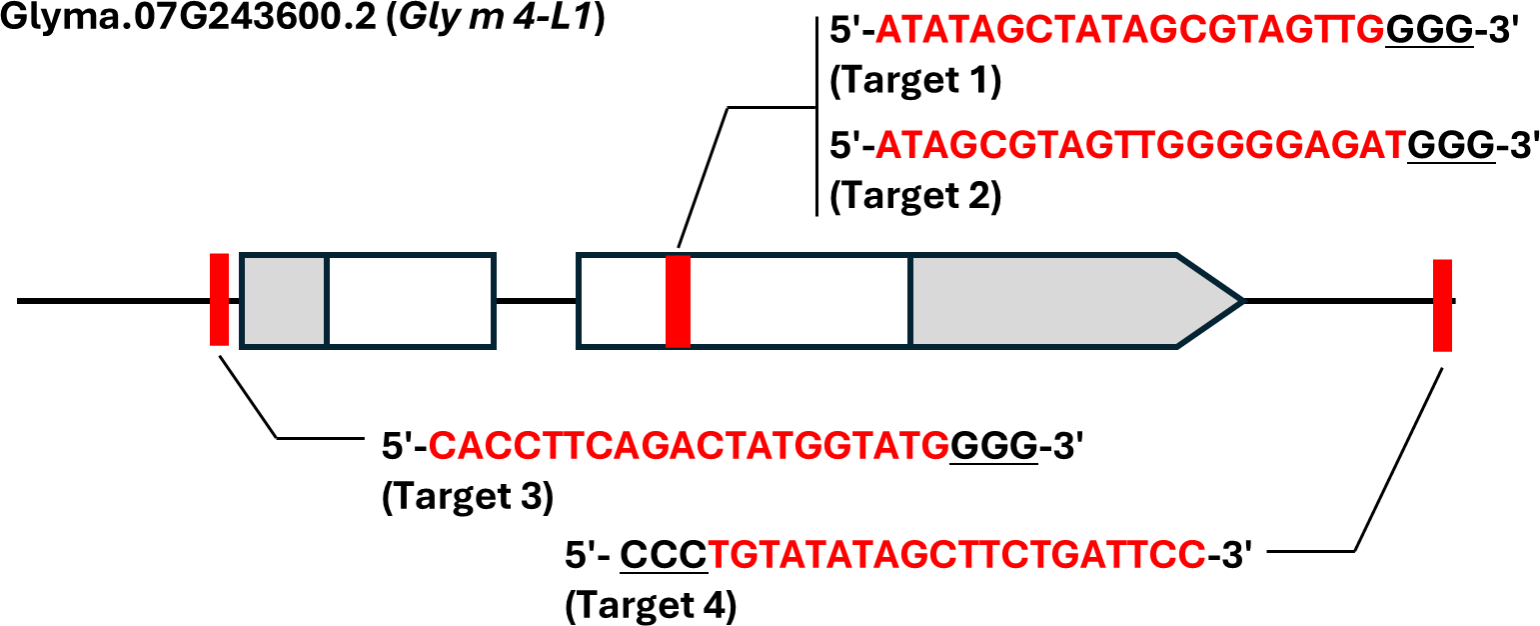
Schematic of site-directed mutagenesis of the *Gly m 4-L1* gene using the iPB-RNP system. Exons are shown as boxes and pentagons, with gray regions representing untranslated sequences; pentagons indicate transcriptional endpoints. Red vertical lines mark gRNA target sites. Sequences of the gRNA target regions are shown, with underlined nucleotides indicating PAMs. Brackets below the sequences denote the names of the respective gRNA target sites.

The strong immunoblot signal detected in the *Gly m 4-2^del^* mutant using a polyclonal anti–Gly m 4 antibody suggested the presence of antibody-reactive proteins encoded by genes other than *Gly m 4-2*. To identify potential source sequences, a protein BLAST search was performed against the Phytozome database. Six homologous genes—*Glyma*.07G243600.2, *Glyma*.07G243900.1, *Glyma*.17G030100.1, *Glyma*.17G030200.1, *Glyma*.17G030300.1, and *Glyma*.17G030400.1—were selected for further investigation, excluding *Gly m 4–1* and *Gly m4-2* (**Supplementary Figure 2**). These homologs shared a 52.5–91.8% amino acid sequence identity with the *Gly m 4–1* and *Gly m 4–2* proteins (**Supplementary Figure 3**). qRT-PCR analysis across various tissues showed that all eight genes exhibited higher expression in seedling roots than in other tissues (**Figure 3**). Notably, *Glyma*.07G243600.2 also showed strong expression in seeds (**Figure 3**; **Supplementary Figure 4**) and was selected as the primary target for further mutagenesis. This gene was designated *Gly m 4-L1*. For site-directed mutagenesis of *Gly m 4-L1*, four CRISPR/Cas9 target sites were designed (**Figure 4**). Target 1 and Target 2 were located within the second exon to induce frameshift mutations, while Target 3 and Target 4 were positioned upstream and downstream of the transcribed region, respectively, to generate a null allele through simultaneous double-strand breaks mediated by Cas9 (**Figure 4**).

### 2.4 Generation of *Gly m 4-L1* mutant plants

**Figure 5:**
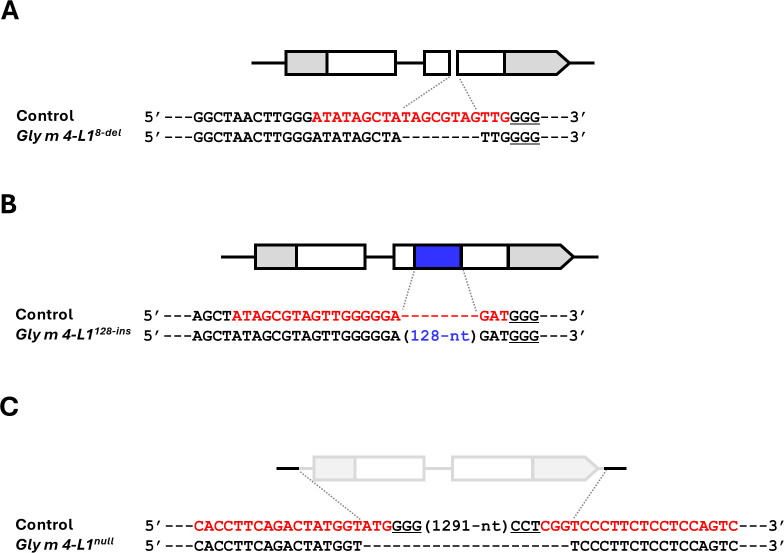
Schematic of mutations in the *Gly m 4-L1* locus. **(A)** Target 1, **(B)** Target 2, and **(C)** simultaneous targeting of Target 3 and Target 4. Exons are represented by boxes and pentagons, with gray regions indicating untranslated sequences; pentagons mark transcriptional endpoints. The blue region denotes the 128-nt insertion, and translucent boxes/pentagons indicate deletions. Red sequences show gRNA target sites, and underlined sequences indicate PAM motifs.

A total of 442, 234, and 522 embryo axes were subjected to site-directed mutagenesis at Target 1, Target 2, and simultaneously at Target 3 and Target 4, respectively (**Table 1**). Mutations at Target 1 and Target 2 in the E_0_ generation were confirmed by *in vitro* RNP assays, while mutations induced by simultaneous cleavage at Target 3 and Target 4 were identified based on PCR amplicon sizes using a specific primer set. Site-directed mutagenesis generated five, two, and six mutant plants for Target 1, Target 2, and the combined Target 3/Target 4 treatments, respectively, in the E_0_ generation (**Table 1**). Mutant alleles were transmitted to the E_1_ generation in four of the 13 E_0_ mutants (**Table 1**). In the E_2_ generation, all mutant lines were analyzed, and segregation was observed in all cases, yielding individuals homozygous for the mutant alleles (**Supplementary Figure 5**). Sequencing of the target regions revealed distinct mutation patterns. For Target 1, E_2_ individuals from two independent embryo axes each carried an identical 8-bp deletion and were designated *Gly m 4-L1^8-del^* mutants (**Figure 5A**). Target 2 mutations consisted of a 128-bp insertion (**Figure 5B**), which BLAST analysis confirmed as matching an intergenic region on chromosome 7 (**Supplementary Figure 6**); this mutant was designated *Gly m 4-L1^128-ins^*. Simultaneous targeting of Target 3 and Target 4 produced a 1,303-nucleotide deletion spanning the entire transcribed region of *Gly m 4-L1*, generating the *Gly m 4-L1^null^* mutant (**Figure 5C**).

**Table 1:** Frequencies of mutation induction at each target site within the *Gly m 4-L1* locus.

Target regions	No. of bombarded embryonic axes	No. of mutant plants in E_0_ generation	No. of mutant plants in E_1_ generation	Frequencies of mutation induction
Target 1	442	5	2	1.1%
Target 2	234	2	1	0.9%
Targets 3 and 4	522	6	1	1.1%

### 2.5 Characterization of *Gly m 4-L1* mutants

**Figure 6:**
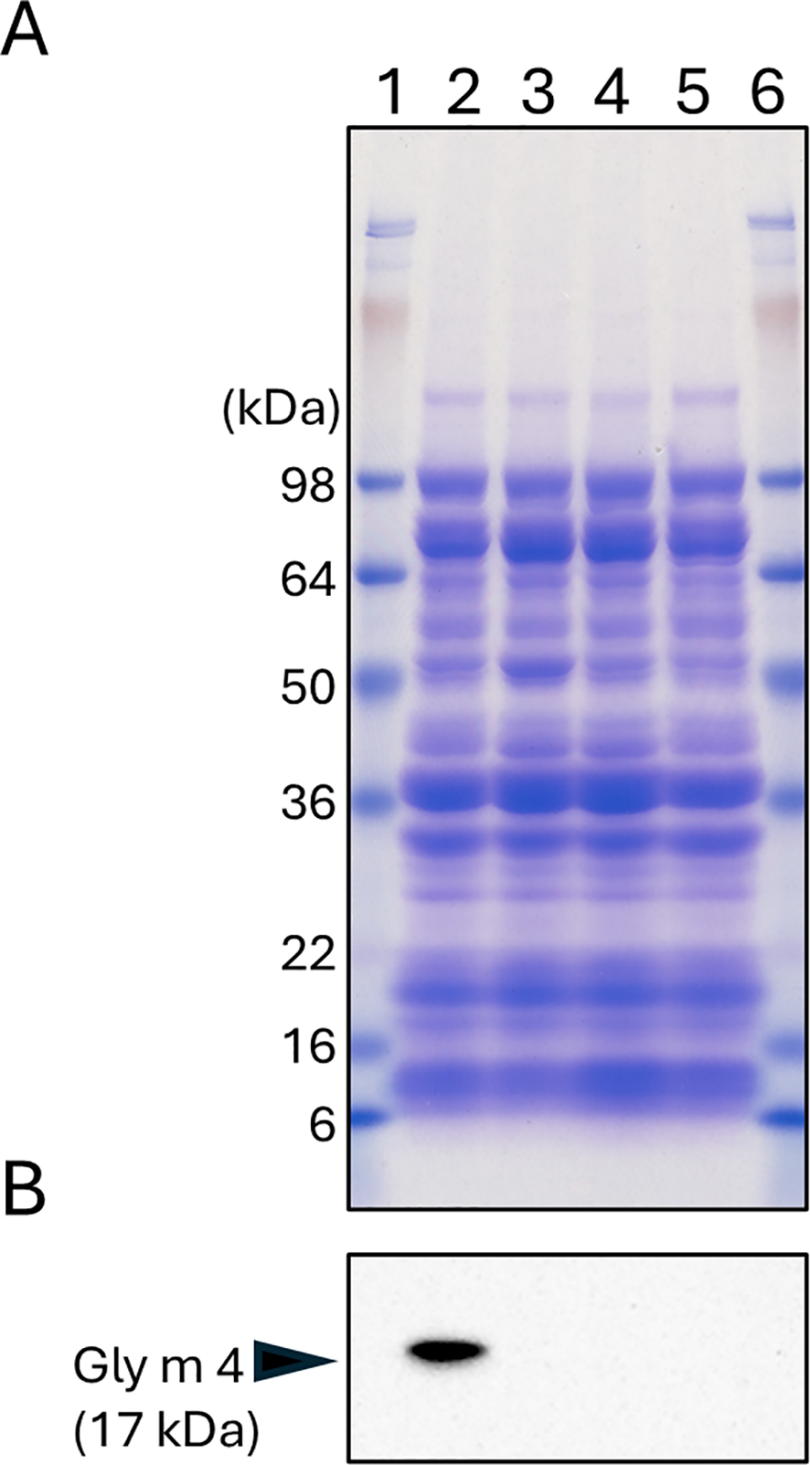
SDS-PAGE and immunoblot analyses of total seed proteins from control and *Gly m 4-L1* mutant lines. **(A)** SDS-PAGE: Lanes 1, protein molecular weight markers; lane 2, control; lane 3, *Gly m 4-L1^8-del^* mutant; lane 4, *Gly m 4-L1^128-ins^*; lane 5, *Gly m 4-L1^null^*. **(B)** Immunoblot probed with polyclonal anti–Gly m 4 antibody.

To assess the impact of each mutation on the amino acid sequence, cDNA from the *Gly m 4-L1^8-del^* and *Gly m 4-L1^128-ins^* mutants was amplified and sequenced (**Supplementary Figure 7**, **8**). Primers were designed to flank the start and stop codons of the wild-type *Gly m 4-L1* transcript. Sequence analysis showed that the *Gly m 4-L1^8-del^* allele produces a truncated open reading frame relative to the wild type (**Supplementary Figure 7**, **8**). In the *Gly m 4-L1^128-ins^* mutant, the 128-nucleotide insertion induced an alternative splicing event at the target locus, generating a shorter coding sequence (**Supplementary Figure 7** and **9**). No transcript was detected in the *Gly m 4-L1^null^* mutant, consistent with a complete loss of expression at this locus (**Supplementary Figure 7**, **8**). SDS-PAGE analysis of crude seed protein extracts revealed similar electrophoretic banding patterns for the control and mutant lines (**Figure 6A**). In contrast, immunoblotting with a polyclonal anti–Gly m 4 antibody detected a strong signal only in the control seeds (**Figure 6B**). Neither the *Gly m 4-L1^8-del^* nor *Gly m 4-L1^128-ins^* mutants displayed Gly m 4-reactive bands or novel immunoreactive species (**Supplementary Figure 10**).

### 2.6 Allergenicity testing of proteins from *Gly m 4-2^del^* and *Gly m 4-L1^null^* mutants

**Figure 7:**
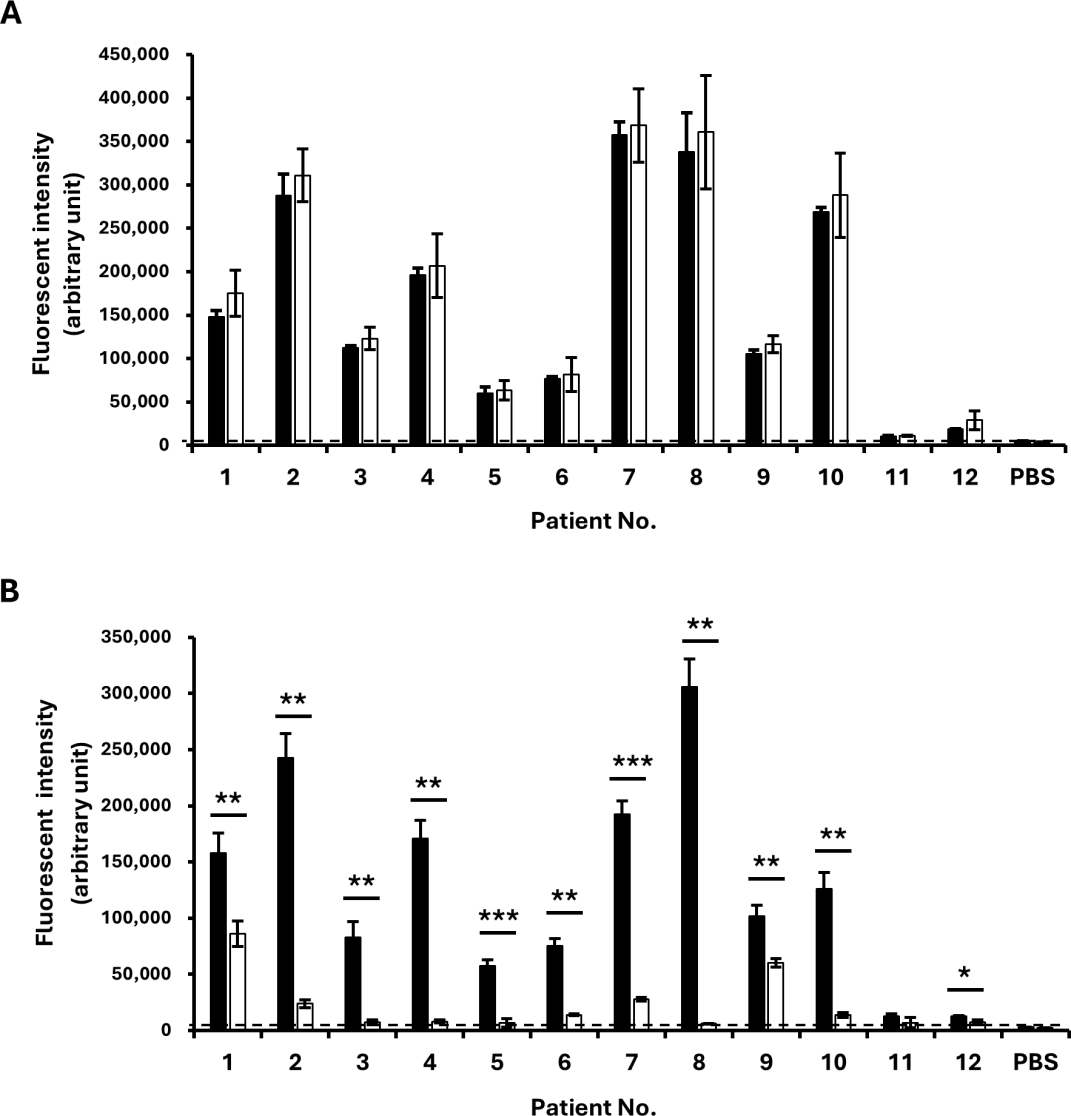
Allergenic assessment of *Gly m 4-2^del^* and *Gly m 4-L1^null^* mutants using sera from Gly m 4–positive patients. ELISA was performed on protein fractions obtained by gel filtration chromatography from seed extracts of control plants, *Gly m 4-2^del^* mutants **(A)**, and *Gly m 4-L1^null^* mutants **(B)** in PBS. Black and white bars represent control and mutant samples, respectively. Data are means ± SD from three independent experiments. Horizontal dotted lines indicate negative control values (PBS without serum). *, **, and *** denote significant differences at the 5%, 1%, and 0.1%, respectively.

Protein fractions were isolated from mature seeds of control, *Gly m 4-2^del^*, and *Gly m 4-L1^null^* mutant plants through gel filtration chromatography (**Supplementary Figure 11A**). To identify fractions containing Gly m 4 proteins, eluates were analyzed by immunoblotting with a polyclonal anti–Gly m 4 antibody (**Supplementary Figure 11B**). Fractions 14–21 from control seeds produced clear Gly m 4 signals and were subsequently selected for allergenicity testing using serum from Gly m 4–positive patients. Patient demographics and clinical data, such as reactivity to birch pollen and Rosaceae fruits, are summarized in **Supplementary Table 1**. ELISA measurements showed no significant difference in serum reactivity between control and *Gly m 4-2^del^* fractions (**Figure 7A**). In contrast, fractions from *Gly m 4-L1^null^* mutant seeds exhibited markedly reduced signal intensities compared with controls (**Figure 7B**), indicating a strong decrease in allergenic potential.

### 2.7 Characterization of off-target mutations in *Gly m 4-L1* mutants

Off-target mutations were evaluated in the *Gly m 4-L1* mutants generated in this study. We sequenced homologous loci within the *Gly m 4-1*, *Gly m 4-2*, and *Glyma.17G030200.1* genes, as well as high-ranking potential off-target sites predicted by CHOPCHOP (https://chopchop.cbu.uib.no/). No off-target mutations were detected in any of the examined regions (**Supplementary Table 2**).

### 2.8 Evaluation of agronomic characteristics of *Gly m 4-L1* mutants

Control, *Gly m 4-L1^8-del^*, *Gly m 4-L1^128-ins^*, and *Gly m 4-L1^null^* mutant plants grown in a greenhouse were assessed for vegetative and seed morphology. No differences in plant architecture or seed characteristics were observed among the genotypes (**Supplementary Figure 12**). Furthermore, seed nitrogen quantification revealed that a significant decrease was observed only in *Gly m 4-L1^128-ins^*, whereas no significant changes were detected in the other mutants (**Supplementary Figure 13**). Based on a nitrogen-to-protein conversion factor of 5.71, the total protein content of the generated *Gly m 4-L1* mutants was estimated to range from 31.9% to 35.5%, compared with 35.9% in the wild-type.

## 3 Discussion

The present study establishes Gly m 4-L1 as the predominant isoform responsible for the allergenicity of soybean seeds. Although Gly m 4 was historically classified as a PR-10 (Graham et al., 2003) and subsequent immunological studies have relied on the P26987 reference sequence, our findings demonstrate that the biochemical properties of seed-derived Gly m 4 are primarily derived from the *Gly m 4-L1* gene product. This is supported by our observation that while *Gly m 4–2* matches the reference sequence, its targeted deletion failed to alter total Gly m 4 levels in seeds. In contrast, *Gly m 4-L1* deficiency resulted in a complete absence of detectable Gly m 4 protein (**Figure 2**, **7**).

All site-directed mutagenesis experiments were conducted using the soybean variety Yukihomare. To determine whether *Gly m 4* expression patterns are conserved across genotypes, we analyzed data from the Daize-net gene expression atlas (https://daizu-net.dna.naro.go.jp/ap/top; Yano et al., 2025). In both Enrei and Williams 82, *Gly m 4-L1* showed the highest seed-specific expression among all *Gly m 4* homologs (**Supplementary Table 3**). Moreover, Julka et al. (2012) purified native Gly m 4 from soybean seeds and reported a sequence identical to *Glyma*.07G243600.2, which corresponds to the *Gly m 4-L1* gene. Collectively, these findings establish *Gly m 4-L1* as the principal seed-expressed gene encoding the Gly m 4 allergen across soybean varieties, and its product underlies the characteristic biochemical and immunological properties of Gly m 4 in seeds.

Allergenicity assays using sera from *Gly m* 4–positive patients enabled detailed immunological profiling of Gly m 4 homologs (Mittag et al., 2004). Protein fractions from *Gly m 4-L1^null^* mutants showed a markedly greater reduction in IgE reactivity than fractions from *Gly m 4-2^del^* mutants (**Figure 7**). In contrast, mutating the minimally expressed *Gly m 4–2* gene did not alter overall allergenicity, consistent with its negligible contribution to Gly m 4 accumulation in seeds (**Figure 7A**). These findings identify *Gly m 4-L1* as the principal allergenic isoform in soybean seeds for individuals sensitized to Gly m 4. Notably, residual ELISA signals varied among patients: sera from patients 1 and 9 exhibited higher reactivity to *Gly m 4-L1^null^*-derived fractions than those from other patients (**Figure 7B**). To explore this variation, control seed proteins were separated using SDS-PAGE, and peptides from bands corresponding to Gly m 4-sized proteins were analyzed using LC-MS/MS. In addition to *Gly m 4-L1*, peptides matching *Glyma*.17G030200.1 and *Glyma*.17G030300.1 were detected, covering 97% of their sequences (**Supplementary Figure 14**). Therefore, the residual IgE reactivity observed in patients 1 and 9 may reflect binding to these low-abundance proteins, which remained below the detection limit of immunoblotting. Simultaneous site-directed mutagenesis of *Gly m 4-L1*, along with *Glyma*.17G030200.1 and *Glyma*.17G030300.1—an “allergen stacking” approach—could further reduce the allergenic potential of soybean seeds. Alternatively, the residual reactivity may arise from IgE recognition of other seed allergens, such as Gly m Bd 28K or Gly m Bd 30K (Ogawa et al., 1991; Tsuji et al., 1997), which are also present in the tested fractions.

Cross-reactivity between Bet v 1 and Gly m 4 is a well-established immunological phenomenon (Kleine-Tebbe et al., 2002). Epitope mapping of Bet v 1 identified residues 142–156 as critical for IgE cross-reactivity with related food allergens (Jahn-Schmid et al., 2005). This region shares an 80% sequence identity with Gly m 4 (UniProt P26987), providing a molecular basis for their cross-reactivity (Jahn-Schmid et al., 2005). Although Gly m 4 adopts a Bet v 1–like fold, subtle conformational differences have been reported (Berkner et al., 2009). Furthermore, Finkina et al. (2022) proposed that multiple surface regions of Gly m 4 may contribute to IgE binding due to the digestive resistance and high structural stability of the protein. In this study, immunoblotting using a polyclonal anti–Gly m 4 antibody detected no signal for proteins from the *Gly m 4-L1^8-del^* and *Gly m 4-L1^128-ins^* mutants (**Figure 6B**), indicating that these variants likely adopt conformations distinct from the wild-type. However, the possibility remains that some IgE-binding regions are retained. In contrast, the *Gly m 4-L1^null^* mutant, which completely lacks the coding sequence, eliminates all Gly m 4 epitopes. Notably, generating this null allele required simultaneous CRISPR/Cas9-mediated cleavage at two sites (Targets 3 and 4); however, the frequency of heritable mutations was comparable to that of single-site edits (Targets 1 or 2) (**Table 1**). This highlights that multiplex deletion of the entire *Gly m 4-L1* locus is both a robust and efficient strategy.

To the best of our knowledge, this study is the first to systematically report on the development of hypoallergenic legume lines and evaluate their reduced allergenicity based on patient-derived immunoreactivity. Nonetheless, there are several concerns that need to be addressed. In the application of the CRISPR/Cas9 system, the occurrence of off-target mutations represents a major obstacle to the practical utility of generated mutants (Manghwar et al., 2019). In this study, no mutations were detected in any of the predicted off-target sites examined (**Supplementary Table 2**). Previous studies have also demonstrated that iPB-RNP-mediated site-directed mutagenesis does not induce off-target mutations (Kumagai et al., 2022; Asa et al., 2025). Notably, the iPB-RNP method employed here introduces RNPs directly into cells, where they are rapidly degraded. This transient presence likely reduces the risk of off-target effects compared to conventional transformation-based methods.

Although *Gly m 4-L1* belongs to the PR-10 protein family, which is frequently associated with disease resistance, its loss of function raises concerns regarding its potential impact on the plant’s overall defense capabilities. Indeed, previous studies have demonstrated the multifaceted roles of PR-10; for instance, overexpression of the *PR-10* gene in transgenic tobacco and soybean enhances resistance to *Phytophthora nicotianae* and *P. sojae*, respectively (Xu et al., 2014). Furthermore, *PR-10* overexpression in pea (*Pisum sativum*) has been shown to modulate the composition of the rhizosphere microbial community (Ruiz-Lozano et al., 1999). However, expression profiling demonstrated that other homologs exhibit higher transcript levels in roots and leaves, organs that are more frequently exposed to pathogen stress than seeds. These findings suggest that these homologs provide functional redundancy or compensation for the deficiency of the *Gly m 4-L1*. Furthermore, phenotypic evaluation revealed no notable differences in agronomic traits or seed nitrogen content between the mutants and control plants under greenhouse conditions (**Supplementary Figure 12**, **13**). These results indicate that the loss of *Gly m 4-L1* does not substantially affect major agricultural traits at least in a greenhouse environment, though further field-based assessments are warranted to evaluate performance under realistic environmental conditions. Importantly, the lack of off-target mutations and the maintenance of stable agronomic traits underscore the practical potential of this line. While field trials are essential to confirm performance under environmental stress, our results demonstrate that functional redundancy by other PR-10 homologs in vegetative tissues likely mitigates the biological cost of seed-specific *Gly m 4-L1* loss.

## 4 Materials and methods

### 4.1 Plant materials

*Glycine max* L. cv. Yukihomare was used in all experiments. Seeds were sown in commercial soil (Katakura and Co-op Agri Corp.) and grown at 25 °C in an isolated greenhouse at Hokkaido University dedicated to transgenic plant research.

### 4.2 Selection of candidate genes for site-directed mutagenesis

The UniProt entry P26987, retrieved from the NCBI database (https://www.ncbi.nlm.nih.gov/), served as the reference sequence for *Gly m 4*. This sequence was used to identify high‐homologous genes in the *Glycine max* Wm82.a6.v1 genome (Phytozome genome ID: 880). Multiple sequence alignments of the retrieved *Gly m 4* homologs were conducted using the ClustalW algorithm implemented in MEGA11 (https://www.megasoftware.net/).

### 4.3 Expression analysis of Gly m 4 and its homologs

Total RNA was extracted from immature and mature seeds and the leaves and roots of juvenile seedlings using the LiCl precipitation method (Adachi et al., 2021). RNA samples were stored at −80 °C until analysis. First-strand cDNA was synthesized from 1 μg of total RNA using M-MLV reverse transcriptase (Invitrogen) with a mixture of random hexamer and oligo(dT)20 primers, following the manufacturer’s instructions. qRT-PCR was performed in 20 μL reactions containing 1 μL of cDNA, 0.8 μL of each 10 μM primer, and 10 μL of SYBR Premix Ex Taq II (Tli RNaseH Plus; TaKaRa). Amplifications were conducted on a CFX96 Real-Time System (Bio-Rad) under the following conditions: 95 °C for 3 min, followed by 40 cycles of 95 °C for 5 s and 60 °C for 30 s. Melting curve analysis confirmed the amplification of a single product. The following genes were analyzed: *Glyma*.07G243500.2 (*Gly m 4-1*), *Glyma*.07G243651.1 (*Gly m 4-2*), *Glyma*.07G243600.2 (*Gly m 4-L1*), *Glyma*.07G243900.1, *Glyma*.17G030100.1, *Glyma*.17G030200.1, *Glyma*.17G030300.1, and *Glyma*.17G030400.1. Expression levels were normalized to 18S rRNA (XR_003264275). Relative expression and standard error were calculated from three biological replicates. Primer sequences are listed in **Supplementary Table 4**.

### 4.4 Design of gRNA and production of mutant soybean plants

A 20-nucleotide sequence targeting the *Gly m 4–2* and *Gly m 4-L1* loci was selected using three online tools: CRISPR-P 2.0 (http://crispr.hzau.edu.cn/CRISPR2/), CHOPCHOP, and CRISPRdirect (https://crispr.dbcls.jp/). For DNA-free site-directed mutagenesis, single guide RNAs (sgRNAs) were assembled with Cas9 protein to form RNP complexes. The sgRNAs were synthesized by FASMAC Co., Ltd., dissolved in nuclease-free water at 1 µg/µL, and stored at −80 °C until use. Preparation of soybean embryonic axes, iPB-RNP, and post-bombardment cultures of explants were performed following Asa et al. (2025). Additionally, these procedures, from particle bombardment to plant regeneration, are described in greater detail with accompanying photographs in our previous report (Kuwabara et al., 2024).

### 4.5 Detection of mutations in target regions

Genomic DNA was extracted from the bulked leaf tissue of bombarded seedlings, with three to four trifoliate leaflets collected from each E_0_ plant. DNA from leaves and mature seeds was used for mutation analysis in the E_1_ generation, following Sugano et al. (2020). Induced mutations were detected either by *in vitro* digestion with CRISPR/Cas9–RNP complexes (Kumagai et al., 2022) or by assessing PCR amplicon sizes using locus‐specific primers (**Supplementary Table 4**).

### 4.6 Sequencing analysis

PCR products spanning the CRISPR target sites were purified using ExoSAP-IT (Affymetrix) and either directly Sanger sequenced or cloned into the pGEM-T Easy vector (Promega) before sequencing. All sequencing analyses were outsourced to FASMAC Co., Ltd.

### 4.7 Production of polyclonal antibodies to Gly m 4

Recombinant Gly m 4 protein was expressed in *Escherichia coli* and purified as previously described by Maruyama et al. (2018). Briefly, the coding sequence of Gly m 4 was cloned into the bacterial expression vector pGEX (Cytiva) and transformed into *E. coli* SHuffle T7 (New England Biolabs). The recombinant protein was purified by affinity chromatography, followed by removal of the GST tag using PreScission protease (Cytiva), and further purified by gel filtration chromatography. The purified recombinant Gly m 4 protein was used as an immunogen to generate polyclonal antibodies in a rabbit. The titer of the resulting polyclonal antiserum against Gly m 4 was confirmed by ELISA.

### 4.8 Immunological analysis of crude soybean seed proteins

E_3_ and E_4_ generation seeds were employed for protein extraction. Mature seeds were pulverized into a fine powder using a Multi-Beads Shocker (MB1200; Yasui Kikai Co.). A 5-mg aliquot of the resulting powder was suspended in 1 mL of protein extraction buffer (50 mM Tris-HCl, pH 8.0, 0.2% SDS, 5 M urea, and 2% 2-mercaptoethanol) and vortexed vigorously for 10 min. Following centrifugation at 14,000 ×g for 2 min, the supernatant (4 μL) was analyzed by SDS-PAGE using a 5–12% gradient gel (ATTO). Crude seed proteins separated by SDS-PAGE were transferred to PVDF membranes (Hybond-P; GE Healthcare). Membranes were blocked overnight at 4 °C with 5% skim milk (Wako) and then probed with anti–Gly m 4 antiserum using the ECL Plus Western Blotting system (GE Healthcare).

### 4.9 Fractionation of soybean seed proteins

Soybean seed powder was suspended in phosphate-buffered saline (PBS) and stirred at room temperature for 30 min to extract proteins. The mixture was centrifuged, and the supernatant was collected and subjected to gel filtration chromatography (HiLoad 16/60 Superdex 200 prep grade, GE Healthcare) pre-equilibrated with PBS. Proteins were eluted with PBS at 0.7 ml/min, and fractions were analyzed by SDS-PAGE and Western blotting using anti–Gly m 4 antiserum to identify Gly m 4–containing fractions. These fractions were subsequently used for ELISA.

### 4.10 Measurement of IgE reactivity to fractions from control and mutant soybean seeds

Sera from 12 soybean-sensitized patients referred to Sagamihara National Hospital were analyzed. The clinical profiles and laboratory findings of all 12 patients suggest that their soy allergy was induced by Betulaceae-related pollen-food allergy syndrome, rather than primary sensitization via oral soy ingestion. This is supported by the consistently elevated levels of Alder-specific IgE across the cohort. Additionally, detailed patient characteristics are summarized in Supplemental **Table 1**. Ethical approval was obtained from the institutional review boards of both the hospital and Kyoto University. ELISA was performed as described by Maruyama et al. (2016), except that gel filtration fractions were used as coating solutions instead of purified protein. Briefly, 96-well plates (PerkinElmer) were coated overnight at 4 °C with the selected fractions, washed, and blocked with 1% BSA. Patient sera were incubated for 3 h at room temperature, followed by incubation with enzyme-labeled anti-human IgE antibody for 2 h. After washing, the substrate solution was added, and the mixture was incubated at 37 °C for 1 h. Subsequently, the reaction was stopped, and fluorescence intensity was measured using a microplate reader (ARVO X2, PerkinElmer).

### 4.11 Off-target analysis

As with *Gly m 4-1*, *Gly m 4-2*, and *Glyma.17G030200.1*, potential off-target sites for the gRNA used in the site-directed mutagenesis of *Gly m 4-L1* were identified using CHOPCHOP. High-scoring potential off-target sites were amplified with specific primers and subjected to direct sequencing analysis. Primer sequences for these assays are summarized in **Supplementary Table 4**.

### 4.12 Determination of total nitrogen in mature seeds

E_4_ generation seeds were employed for measurement of nitrogen. For each line, three to five mature seeds were ground to a fine powder using a Multi-beads Shocker. The nitrogen content of the powder were measured with a UNICUBE elemental analyzer (Elementar). Total protein content was calculated by multiplying the total nitrogen content by a conversion factor of 5.71, which is specific to soybean protein.

### 4.13 Statistical analysis

Data were analyzed using the Real Statistics Resource Pack for Microsoft Excel (https://real-statistics.com/free-download/real-statistics-resource-pack/). For two-group comparisons, Student’s *t*-tests were utilized. For multiple comparisons, Tukey’s HSD tests were performed following one-way analysis of variance (ANOVA). A *p*-value of less than 0.05 was considered statistically significant.

## 5 Conclusion

Expression profiling of the eight *Gly m 4* homologs identified *Gly m 4-L1* as the primary target for mutagenesis due to its predominant expression in seeds. By delivering an RNP complex, comprising *Gly m 4-L1*-specific gRNA(s) and Cas9 protein, into the shoot apical meristem using the iPB-RNP method successfully generated mutant alleles at the *Gly m 4-L1* locus, several of which were stably inherited across generations. Both indel and null mutations at this locus abolished the accumulation of the wild-type Gly m 4*-*L1 protein in mature seeds. Protein fractions from the *Gly m 4-L1^null^* mutant seeds exhibited markedly reduced IgE reactivity compared with controls. Notably, the mutant lines exhibited no changes in plant architecture or seed morphology. These results demonstrate that targeted knockout of *Gly m 4-L1* using the iPB-RNP approach effectively generates hypoallergenic soybean plants.

## Data Availability

The original contributions presented in the study are included in the article/Supplementary Material. Further inquiries can be directed to the corresponding author.
