# Supplementary material for: CRISPR/Cas9 ribonucleoprotein-mediated knockout of Gly m 4-L1 eliminates allergen accumulation in soybean
Source: Front Plant Sci. 2026 Mar 9;17:1739979. doi: 10.3389/fpls.2026.1739979 (PMC13006505; doi:10.3389/fpls.2026.1739979)
Supplement: Supplementary file 1 [file Table1.docx]

Supplemental Table 1. Demographics, symptoms following soy ingestion and the levels of IgE antibodies in Gly m 4–positive patients

|  | **Gly m 4–positive patients**  **(n=12)** |
| --- | --- |
| Sex (female), n (%) | 9 (75) |
| Age (y), mean ± SD | 39.9 ± 17.8 |
| Allergic comorbidity, n (%)  Allergic rhinitis  Asthma  Atopic dermatitis | 12 (100)  3 (25)  2 (17) |
| Symptoms following soy ingestion, n (%)  Oropharyngeal symptoms  Cutaneous symptoms  Respiratory symptoms  Gastrointestinal symptoms  Cardiovascular symptoms (e.g., hypotension) | 10 (83)  3 (25)  4 (33)  1 (8)  0 (0) |
| Soy products previously reported to provoke symptoms, n (%)  Soy milk  Tofu  Bean sprouts  Edamame (green soybeans)  Soy protein beverages | 10 (83)  5 (42)  2 (17)  1 (8)  1 (8) |
| Oropharyngeal symptoms after fruit consumptions, n (%)  Apple  Peach | 6 (50)  8 (67) |
| Allergen specific IgE antibodies, kUA/l, median (range)*  Gly m 4-IgE  Alder-IgE | 20.3 (3.65-46.7)  36.2 (8.08-93.7) |
| Positive in skin prick test using soybean extract, n (%)** | 9 (100) |

*, ImmunoCAP method

**, For the three patients, a skin prick test using soybean extract was not performed.
