# Supplementary material for: CRISPR/Cas9 ribonucleoprotein-mediated knockout of Gly m 4-L1 eliminates allergen accumulation in soybean
Source: Front Plant Sci. 2026 Mar 9;17:1739979. doi: 10.3389/fpls.2026.1739979 (PMC13006505; doi:10.3389/fpls.2026.1739979)
Supplement: Supplementary file 2 [file Table2.docx]

**Supplementary Table S3.** Evaluation of off-target mutations in *Gly m 4-L1* mutants.

| Predicted off-target locations | Examined mutants | Genomic region | Potential off-target sequences^a^ | No. of mismatch bases | Detection of off-targets |
| --- | --- | --- | --- | --- | --- |
| Target 1 |  |  |  |  |  |
| Gm04:26445207 | *Gly m 4-L1^8-del^*, *Gly m 4-L1^128-ins^* | Intergenic | CCACAACTACagTATAGCTATgT | 3 | Not detected |
| Gm06:650709 | *Gly m 4-L1^8-del^*, *Gly m 4-L1^128-ins^* | Intron | CCCCAAaTAtGCTATAGCTAgAT | 3 | Not detected |
| Gm06:43063692 | *Gly m 4-L1^8-del^*, *Gly m 4-L1^128-ins^* | Intergenic | CCCCAACTACaaTATAGCTATgT | 3 | Not detected |
| Gm07:44901158 | *Gly m 4-L1^8-del^*, *Gly m 4-L1^128-ins^* | Exon | CCCCAACcACGCTgTAGCTgTAT | 3 | Not detected |
| Gm16:31711298 | *Gly m 4-L1^8-del^*, *Gly m 4-L1^128-ins^* | Intergenic | ATATAGaTATAGCaTAcTTGAGG | 3 | Not detected |
| Target 2 |  |  |  |  |  |
| Gm17:2228271 | *Gly m 4-L1^8-del^*, *Gly m 4-L1^128-ins^* | Exon | CCCAaCTCCaCCAACTACGCTAT | 2 | Not detected |
| Gm17:2231346 | *Gly m 4-L1^8-del^*, *Gly m 4-L1^128-ins^* | Exon | ATAGCGTAGTTGGtGGAGtTGGG | 2 | Not detected |
| Target 3 |  |  |  |  |  |
| Gm07:44905440 | *Gly m 4-L1^null^* | Exon | CAgaTTCAcACTATGGTATGGGG | 3 | Not detected |
| Target 4 |  |  |  |  |  |
| Gm12:38733225 | *Gly m 4-L1^null^* | Intron | aGAATCAtAAGCTATATAtATGG | 3 | Not detected |
| Gm14:16015672 | *Gly m 4-L1^null^* | Intergenic | aGAATCAtAAGCTATATAtATGG | 3 | Not detected |

Potential off-target loci were selected by the gRNA design program of CHOPCHOP (https://chopchop.cbu.uib.no/).

^a^Lowercase and underlined nucleotides denote mismatch nucleotides to the target site of gRNA and PAM sequence, respectively.
