# Supplementary material for: CRISPR/Cas9 ribonucleoprotein-mediated knockout of Gly m 4-L1 eliminates allergen accumulation in soybean
Source: Front Plant Sci. 2026 Mar 9;17:1739979. doi: 10.3389/fpls.2026.1739979 (PMC13006505; doi:10.3389/fpls.2026.1739979)
Supplement: Supplementary file 4 [file Table4.docx]

Supplemental Table 4. Primer sequences used for gene expression, cloning, and sequencing analyses.

| Primer name | Primer sequence (5'-3') | Use of amplicon |
| --- | --- | --- |
| 18SrRNA_Fw | TGATTAACAGGGACAGTCGG | Gene expression analysis |
| 18SrRNA_Rv | ACGGTATCTGATCGTCTTCG | Gene expression analysis |
| 3500_Fw | CAAGGAAATTGATACCAGAGGTAG | Gene expression analysis |
| 3500_Rv | GATTATATGTAGAACGGAATGGAAG | Gene expression analysis |
| 3651_Fw2 | CTCCAAATTGGTTGCTGGTC | Gene expression analysis |
| 3651_Rv2 | GTCTCCAAGCAAGAAAGTGATG | Gene expression analysis |
| 3600_Fw | CTGATCCTCTTCATCACTATTCAGTCA | Gene expression analysis |
| 3600_Rv | GCACACACCACACACAGTGA | Gene expression analysis |
| 3900_Fw | GTTGTGGAGTCATACCTGCTAGAG | Gene expression analysis |
| 3900_Rv | CCCTATATTTGGTTGCACACTGC | Gene expression analysis |
| 17G100_Fw | GGCTATTGAGGATTTCATTCAGGC | Gene expression analysis |
| 17G100_Rv | GCAACACACGACAAGAAGGG | Gene expression analysis |
| 17G200_Fw | CGATCTTCAGTATTCAGTGATCTGC | Gene expression analysis |
| 17G200_Rv | CCACACCACACACTCAAAGTGA | Gene expression analysis |
| 17G300_Fw | GCCAATCCTCATTACAACTGATCC | Gene expression analysis |
| 17G300_Rv | GGAAACACTCATTACATTGAAAGCC | Gene expression analysis |
| 17G400_Fw2 | TTTGGCCAATCCCGATTAC | Gene expression analysis |
| 17G400_Rv2 | GGTTTACGTGAGTATGGACTATTA | Gene expression analysis |
| m4-PCR6-Fw | GAGACAAGAAAGACCGCCTAAA | Gene cloning and sequencing analyses |
| m4-PCR5-Rv | CGCCATAAAGAAGGATTCCA | Gene cloning and sequencing analyses |
| 3600-cloning2-Fw | GAATGCGGACAGATGGAGAAAC | Gene cloning and sequencing analyses |
| 3600-cloning2-Rv | CCTCAACGGCCTTGAAAAGAG | Gene cloning and sequencing analyses |
| 3600_seq_Fw | CACAGCAGCAAACATCTCCTTC | Gene cloning and sequencing analyses |
| 128in_Fw | AAGTCTTACCTAGGATCAGG | Gene cloning and sequencing analyses |
| 128in_Rv | ATACTAACTTGTTGACACGTG | Gene cloning and sequencing analyses |
| Gm04-26445207Fw | CTAGGTGCTAATGGGCCCTAC | Off-target analysis |
| Gm04-26445207Rv2 | TGTCGTTTCTCGCTCCATGTTC | Off-target analysis |
| Gm06-650709Fw | CTGCTCATCCCCTTCTGCTCTA | Off-target analysis |
| Gm06-650709Rv | TGAAATGGTCAGATCTCTGAATCC | Off-target analysis |
| Gm06-43063692Fw | ACCCATTAACTGAACCATGACCC | Off-target analysis |
| Gm06-43063692Rv | CCAGAGTGCATAAACTTTGTACTAAT | Off-target analysis |
| Gm16-31711298Fw2 | GTGGGTAGCATCCACTCATAAGAG | Off-target analysis |
| Gm16-31711298Rv | GATAATAACTGAGAGGGAGCTGC | Off-target analysis |
| Gm14-16015672Fw2 | CACAAACAAAATGGGGTTACTA | Off-target analysis |
| Gm14-16015672Rv | GTAGGGCAAACACACACATGAT | Off-target analysis |
| Gm12-38733225Fw | GTGTCTGATCCTGATCTCTGGA | Off-target analysis |
| Gm12-38733225Rv | ATTGGCTCACAGAAGTTGTGATAG | Off-target analysis |
| Gm07-44901158Fw | AGCTTCATTCACACTATGGGTCAT | Off-target analysis |
| Gm07-44901158Rv | GTCTCCAAGCAAGAAAAAGCAAG | Off-target analysis |
| m4-F | TAGGGACCAATGTGTCAGTCGC | Off-target analysis |
| Gm07-44905Fw | GATAATCCAAGTGGAGAAAACCA | Off-target analysis |
| Gm07-44905Rv | GTCTCCAAGCAAGAAAGTGATG | Off-target analysis |
| 07G3651seq-Rv | CACCCATTATGAATGGAAGAATAAT | Off-target analysis |
| Gm17-2228271Fw | GTATTGGGGCCTACATAGAACTAG | Off-target analysis |
| Gm17-2228271Rv | CAGATCACTGAATACTGAAGATCG | Off-target analysis |
| Gm17-2231346Fw | CTATGGGGCATAGAATACAAGGC | Off-target analysis |
| Gm17-2231346Rv | CCAACTTAAAACTTGAGCACAAAG | Off-target analysis |
| 17G200300off-Fw | GATAATGAGGCTGATCATTGG | Off-target analysis |
| 17G200300off-Rv | CAGTTGTAATGAGGATTGGCC | Off-target analysis |
