# Supplementary material for: CRISPR/Cas9 ribonucleoprotein-mediated knockout of Gly m 4-L1 eliminates allergen accumulation in soybean
Source: Front Plant Sci. 2026 Mar 9;17:1739979. doi: 10.3389/fpls.2026.1739979 (PMC13006505; doi:10.3389/fpls.2026.1739979)
Supplement: Supplementary file 5 [file DataSheet1.pdf]

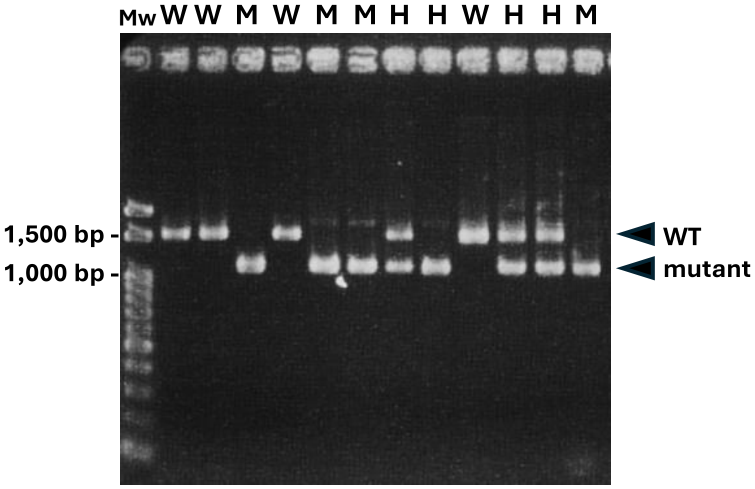

Figure S1. Detection of mutations in the *Gly m 4-2* locus of E_2 progenies of the *Gly m 4-2*^{del} mutant by PCR analysis. The mutant allele yields a shorter amplification product than the wild-type allele. M, H, and W indicate homozygous mutant, heterozygous, and homozygous wild-type genotypes, respectively. Mw denotes the molecular weigh marker.

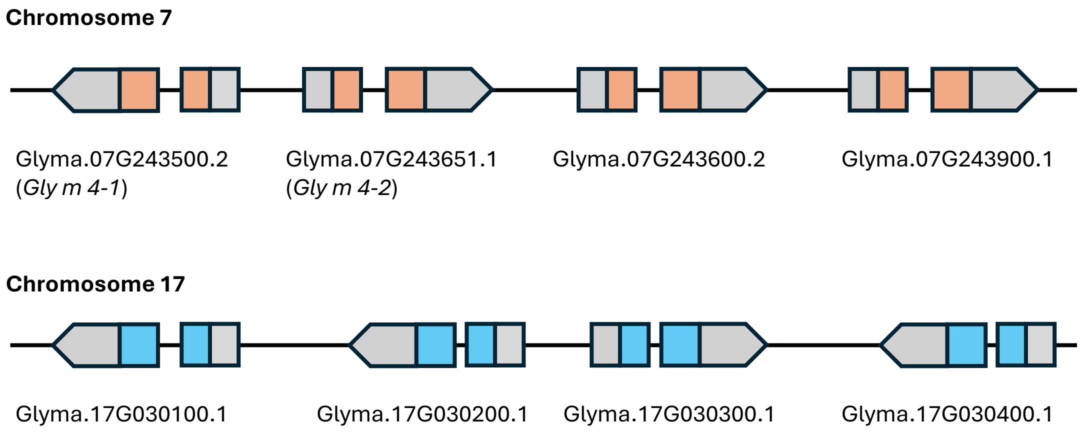

Figure S2. *Gly m 4* homologs identified based on amino acid sequence similarity. Exons are shown as boxes, and untranslated regions are shaded gray. Pentagons indicate transcriptional endpoints. Gene identifiers correspond to annotations from *Glycine max* Wm82.a6.v1, retrieved from Phytozome 13 (https://phytozome-next.jgi.doe.gov/). Homologs encoding amino acid sequences identical to the known Gly m 4 protein (UniProt ID: P26987) are designated *Gly m 4-1* and *Gly m 4-2*.

```
               ....|....| ....|....| ....|....| ....|....| ....|....|
                      10         20         30         40         50
P26987         MGVFTFEDEI NSPVAPATLY KALVTDADNV IPKALDSF-K SVENVEGNGG
07G243500.2    MGVFTFEDEI NSPVAPATLY KALVTDADNV IPKALDSF-K SVENVEGNGG
07G243651.1    MGVFTFEDEI NSPVAPATLY KALVTDADNV IPKALDSF-K SVENVEGNGG
07G243600.2    MGVFTFEDET TSPVAPATLY KALVTDADNV IPKAVDAF-R SVENVEGNGG
07G243900.1    MGITTFTQEY SSSVAPSRMF KALIVDSRNL LPKLLPQFVK DVNVIQGDGE
17G030100.1    MGVFTSESEH VSPVSAAKLY KAIVLDASNV FPKALPNFIK SVETIEGDGG
17G030200.1    MGIFTFEDET TSPVAPATLY KALVTDADNV IPKAVEAF-R SVENLEGNGG
17G030300.1    MGVFTFEDET TSPVAPATLY KALVTDADNV IPKAVDAF-R SVENLEGNGG
17G030400.1    MGIFTFEDEI TSPVAPATLY KALVTDADNI IPKALDSF-K SVENVEGNGG

               ....|....| ....|....| ....|....| ....|....| ....|....|
                      60         70         80         90        100
P26987         PGTIKKITFL EDGETKFVLH KIESIDEANL GYSYSVVGGA ALPDTAEKIT
07G243500.2    PGTIKKITFL EDGETKFVLH KIESIDEANL GYSYSVVGGA ALPDTAEKIT
07G243651.1    PGTIKKITFL EDGETKFVLH KIESIDEANL GYSYSVVGGA ALPDTAEKIT
07G243600.2    PGTIKKITFL EDGETKFVLH KIEAIDEANL GYSYSVVGGD GLPDTVEKIT
07G243900.1    AGSIEQVNFN EDNPFKYLKH RIDVLDKDNL VCKYTMIEGD PLGDKLESIG
17G030100.1    PGTIKKLTLA EG--LGYVKH HVDAIDTENY VYNYSVIEGS ALSEPLEKIC
17G030200.1    PGTIKKITFV EDGESKFVLH KIESVDEANL GYSYSVVGGV GLPDTVEKIT
17G030300.1    PGTIKKITFV EDGESKFVLH KIESVDEANL GYSYSVVGGV GLPDTVEKIT
17G030400.1    PGTIKKITFV EDGETKFVLH KIEAVDEANL GYSYSVVGGA ALPDTAEKIT

               ....|....| ....|....| ....|....| ....|....| ....|....|
                     110        120        130        140        150
P26987         FDSKLVAGPN GGSAGKLTVK YETKGDAEPN QDELKTGKAK ADALFKAIEA
07G243500.2    FDSKLVAGPN GGSAGKLTVK YETKGDAEPN QDELKTGKAK ADALFKAIEA
07G243651.1    FDSKLVAGPN GGSAGKLTVK YETKGDAEPN QDELKTGKAK ADALFKAIEA
07G243600.2    FECKLAAGAN GGSAGKLTVK YQTKGDAQPN QDDLKIGKAK SDALFKAVEA
07G243900.1    YEVKFEATSD GGCLCKMTSN YNTIGEFDVK EEEVKEGRES GIAVYRVVES
17G030100.1    YEYKLVATPD GGSIVKSTSK YYTKGDEQLA EEYVKTGKER SAGFTKAIED
17G030200.1    FECKLAAGAN GGSAGKLTVK YQTKGDAQPN PDDLKIGKVK SDALFKAVEA
17G030300.1    FECKLAAGAN GGSAGKLTVK YQTKGDAQPN PDDLKIGKVK SDALFKAVEA
17G030400.1    FHSKLAAGPN GGSAGKLTVE YQTKGDAQPN QDQLKTGKAK ADALFKAIEA

               ....|....|
                     160
P26987         YLLAHPDYN-
07G243500.2    YLLAHPDYN-
07G243651.1    YLLAHPDYN-
07G243600.2    YLLAHPDYN-
07G243900.1    YLLENPQVYA
17G030100.1    FIQANPDYN-
17G030200.1    YLLANPHYN-
17G030300.1    YLLANPHYN-
17G030400.1    YLLANPDYN-
```

Figure S3. Amino acid sequence similarity between Gly m 4 and its homologues. Gene identifers were obtained from the *Glycine max Wm82.a6.v1* genome assembly, as retrieved from the Phytozome 13 (https://phytozome-next.jgi.doe.gov/). The numbers shown above each sequence indicate the total number of amino acid residues from the start codon. The entry P26987 referred to the Gly m 4 protein sequence registered in the database (https://www.ncbi.nlm.nih.gov/).

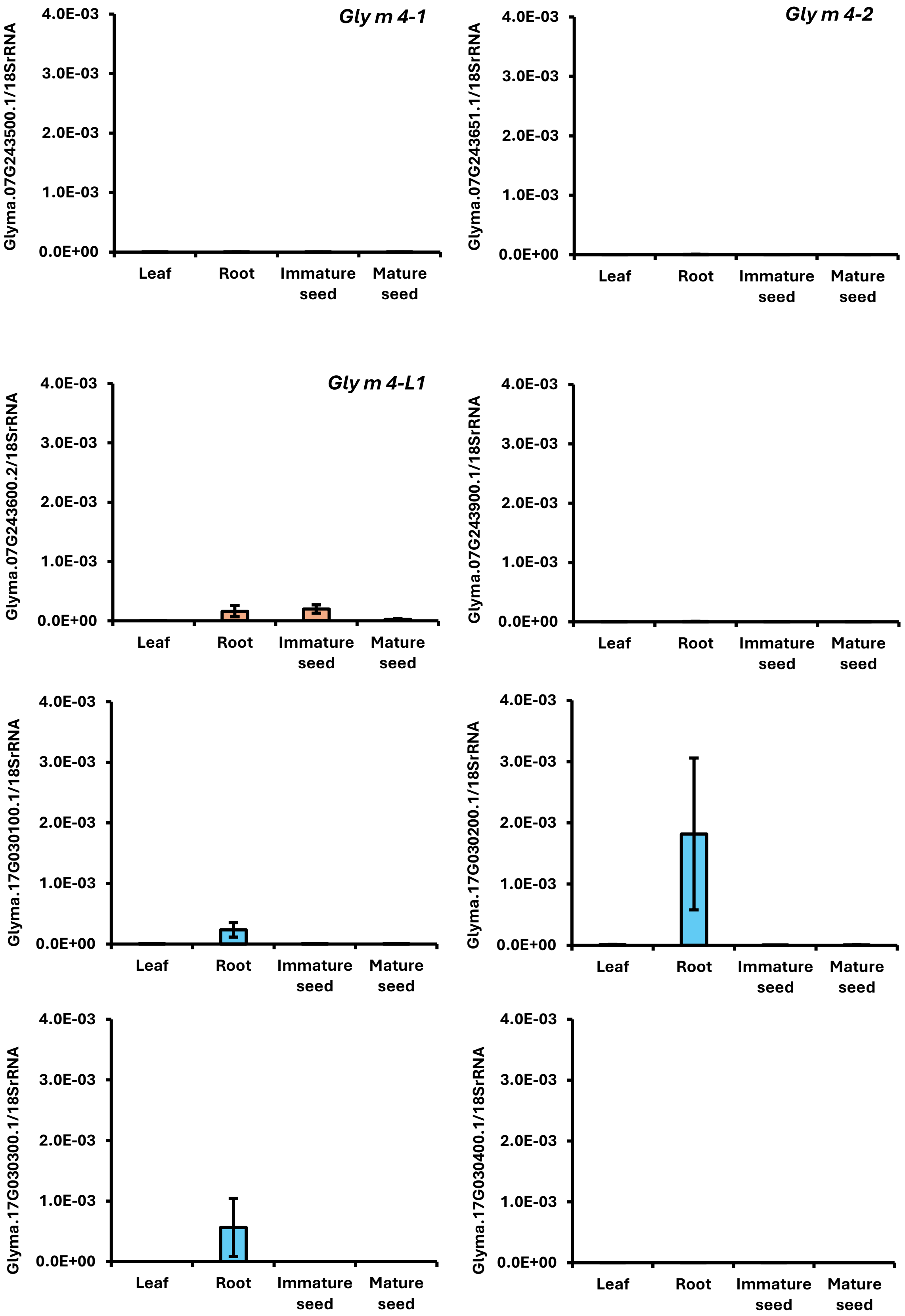

Figure S4. Expression level of Gly m 4 and its homologues in Yukihomare measured by qRT-PCR . Gene identifiers correspond to the *Glycine max Wm82.a6.v1 annotations* from Phytozome 13 (https://phytozome-next.jgi.doe.gov/). The gene targeted for site-directed mutagenesis (Glyma.07G243600.2) was designated as *Gly m 4-L1*. Relative expression was normalized to 18S rRNA reference gene (XR_003264275). Data are means ± SE from three biological replicates.

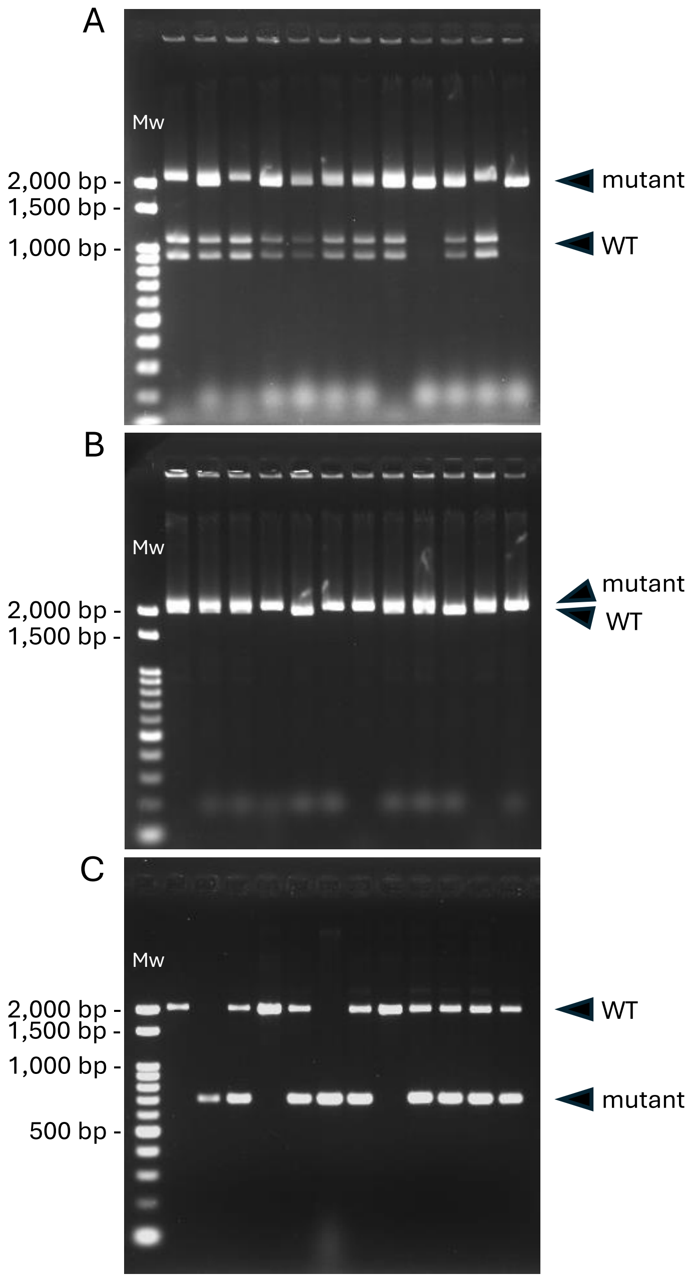

Figure S5. Detection of mutations in E_2 progenies of the *Gly m 4-L1* mutants. Electrophoresis images show genetic segregation pattern in the E_2 generation of three mutant alleles: an 8-nt deletion (**A**), a 128-nt insertion (**B**), and a null mutation (**C**). Panels (**A**) and (**B**) were analyzed by *in vitro* digestion using RNP complexes. Panel (**C**) was evaluated based on the size of PCR products amplified using a specific primer set.

```
Query     1        TTTTTTACATCTTGATCTCCTTAATTTAAAGAGTTTTAATTTAGTCTATTAATTAAATTA  60
                   ||||||||||||||||||||||||||||||||||||||||||||||||||||||||||||
Subject   6043618  TTTTTTACATCTTGATCTCCTTAATTTAAAGAGTTTTAATTTAGTCTATTAATTAAATTA  6043677

Query     61       AACTAACCATTTTATCATATATTTTTATTAATGATGTTAATTTGAGCTGACATAATAAAC  120
                   ||||||||||||||||||||||||||||||||||||||||||||||||||||||||||||
Subject   6043677  AACTAACCATTTTATCATATATTTTTATTAATGATGTTAATTTGAGCTGACATAATAAAC  6043737

Query     121      ATTCGTCT  128
                   ||||||||
Subject   6043738  ATTCGTCT  6043745
```

Figure S6. Alignment of 128-nucleotide sequence inserted at the Target 2 site with a homologous region in the genome soybean genome. The query sequence corresponds to the 128-nucleotide fragment inserted at the Target-2 site. Homologous sequences were retrieved from the soybean genome database (Phytozome 13; https://phytozome-next.jgi.doe.gov/). Numbers shown on both sides of the subject sequences indicate the physical positions on chromosome 7, based on the *Glycine max Wm82.a6.v1* genome assembly.

```
             ....|....| ....|....| ....|....| ....|....| ....|....| ....|....|
                     10         20         30         40         50         60
07G243600.2  ATGGGTGTTT TCACATTCGA GGATGAAACC ACCTCTCCTG TGGCTCCTGC TACCCTTTAC
8-del        ATGGGTGTTT TCACATTCGA GGATGAAACC ACCTCTCCTG TGGCTCCTGC TACCCTTTAC
128-ins      ATGGGTGTTT TCACATTCGA GGATGAAACC ACCTCTCCTG TGGCTCCTGC TACCCTTTAC
null         ---------- ---------- ---------- ---------- ---------- ----------

             ....|....| ....|....| ....|....| ....|....| ....|....| ....|....|
                     70         80         90        100        110        120
07G243600.2  AAAGCTCTAG TTACGGATGC TGACAACGTC ATCCCAAAGG CTGTTGACGC CTTCAGGAGT
8-del        AAAGCTCTAG TTACGGATGC TGACAACGTC ATCCCAAAGG CTGTTGACGC CTTCAGGAGT
128-ins      AAAGCTCTAG TTACGGATGC TGACAACGTC ATCCCAAAGG CTGTTGACGC CTTCAGGAGT
null         ---------- ---------- ---------- ---------- ---------- ----------

             ....|....| ....|....| ....|....| ....|....| ....|....| ....|....|
                    130        140        150        160        170        180
07G243600.2  GTTGAAAACG TTGAGGGAAA TGGTGGCCCC GGAACCATCA AGAAGATCAC TTTCCTTGAG
8-del        GTTGAAAACG TTGAGGGAAA TGGTGGCCCC GGAACCATCA AGAAGATCAC TTTCCTTGAG
128-ins      GTTGAAAACG TTGAGGGAAA TGGTGGCCCC GGAACCATCA AGAAGATCAC TTTCCTTGAG
null         ---------- ---------- ---------- ---------- ---------- ----------

             ....|....| ....|....| ....|....| ....|....| ....|....| ....|....|
                    190        200        210        220        230        240
07G243600.2  GATGGAGAAA CCAAGTTTGT GTTGCACAAA ATAGAAGCGA TTGATGAGGC TAACTTGGGA
8-del        GATGGAGAAA CCAAGTTTGT GTTGCACAAA ATAGAAGCGA TTGATGAGGC TAACTTGGGA
128-ins      GA-------- ---------- ---------- ---------- ---------- ----------
null         ---------- ---------- ---------- ---------- ---------- ----------

             ....|....| ....|....| ....|....| ....|....| ....|....| ....|....|
                    250        260        270        280        290        300
07G243600.2  TATAGCTATA GCGTAGTTGG GGGAGATGGG TTGCCAGACA CAGTGGAGAA GATCACATTC
8-del        TATAGCTAT- -------TGG GGGAGATGGG TTGCCAGACA CAGTGGAGAA GATCACATTC
128-ins      ---------- ---------- ---------- --------CA CAGTGGAGAA GATCACATTC
null         ---------- ---------- ---------- ---------- ---------- ----------

             ....|....| ....|....| ....|....| ....|....| ....|....| ....|....|
                    310        320        330        340        350        360
07G243600.2  GAATGCAAAT TGGCTGCTGG CGCCAACGGA GGGTCTGCTG GGAAGCTAAC TGTCAAATAC
8-del        GAATGCAAAT TGGCTGCTGG CGCCAACGGA GGGTCTGCTG GGAAGCTAAC TGTCAAATAC
128-ins      GAATGCAAAT TGGCTGCTGG CGCCAACGGA GGGTCTGCTG GGAAGCTAAC TGTCAAATAC
null         ---------- ---------- ---------- ---------- ---------- ----------

             ....|....| ....|....| ....|....| ....|....| ....|....| ....|....|
                    370        380        390        400        410        420
07G243600.2  CAAACCAAAG GAGATGCTCA GCCCAACCAA GACGACCTCA AAATTGGCAA AGCCAAGTCT
8-del        CAAACCAAAG GAGATGCTCA GCCCAACCAA GACGACCTCA AAATTGGCAA AGCCAAGTCT
128-ins      CAAACCAAAG GAGATGCTCA GCCCAACCAA GACGACCTCA AAATTGGCAA AGCCAAGTCT
null         ---------- ---------- ---------- ---------- ---------- ----------

             ....|....| ....|....| ....|....| ....|....| ....|....| ....|..
                    430        440        450        460        470
07G243600.2  GATGCTCTTT TCAAGGCCGT TGAGGCTTAC CTTTTGGCCC ATCCTGATTA CAACTGA
8-del        GATGCTCTTT TCAAGGCCGT TGAGGCTTAC CTTTTGGCCC ATCCTGATTA CAACTGA
ins-128      GATGCTCTTT TCAAGGCCGT TGAGGCTTAC CTTTTGGCCC ATCCTGATTA CAACTGA
null         ---------- ---------- ---------- ---------- ---------- -------
```

Figure S7. Transcript sequences of the *Gly m 4-L1* locus in control and mutant plants. Shown are transcript sequences of the *Gly m 4-L1* locus from control plants (07G243600.2), and in three mutant lines: *Gly m 4-L1*$^{8\text{-}del}$ (*8-del*)1, *Gly m 4-L1*$^{128\text{-}ins}$ (*128-ins*), and *Gly m 4-L1*^{null} (*null*). The codons 'ATG' and 'TGA', highlighted in red, represent the predicted start and stop codons, respectively.

```
             ....|....| ....|....| ....|....| ....|....| ....|....|
                     10         20         30         40         50
07G243600.2  MGVFTFEDET TSPVAPATLY KALVTDADNV IPKAVDAFRS VENVEGNGGP
8-del        MGVFTFEDET TSPVAPATLY KALVTDADNV IPKAVDAFRS VENVEGNGGP
128-ins      MGVFTFEDET TSPVAPATLY KALVTDADNV IPKAVDAFRS VENVEGNGGP
null         ---------- ---------- ---------- ---------- ----------

             ....|....| ....|....| ....|....| ....|....| ....|....|
                     60         70         80         90        100
07G243600.2  GTIKKITFLE DGETKFVLHK IEAIDEANLG YSYSVVGGDG LPDTVEKITF
8-del        GTIKKITFLE DGETKFVLHK IEAIDEANLG YSYWGRWVAR HSGEDHIRMQ
128-ins      GTIKKITFLE D--------- ---------- ---------- ---TVEKITF
null         ---------- ---------- ---------- ---------- ----------

             ....|....| ....|....| ....|....| ....|....| ....|....|
                    110        120        130        140        150
07G243600.2  ECKLAAGANG GSAGKLTVKY QTKGDAQPNQ DDLKIGKAKS DALFKAVEAY
8-del        IGCWRQRRVC WEANCQIPNQ RRCSAQPRRP QNWQSQV--- ----------
128-ins      ECKLAAGANG GSAGKLTVKY QTKGDAQPNQ DDLKIGKAKS DALFKAVEAY
null         ---------- ---------- ---------- ---------- ----------

             ....|....|
                    160
07G243600.2  LLAHPDYN
8-del        --------
128-ins      LLAHPDYN
null         --------
```

Figure S8. Predicted amino acid sequence of transcripts in control and mutant plants. Shown are the predicted amino acid sequences translated from transcript sequences of the *Gly m 4L-1* locus in control plants (07G243600.2), and in three mutant lines: *Gly m 4-L1*$^{8\text{-}del}$ (*8-del*)1, *Gly m 4-L1*$^{128\text{-}ins}$ (*128-ins*), and *Gly m 4-L1*^{null} (*null*). The numbers of top on sequences indicate the number of amino acid residues from the start codon.

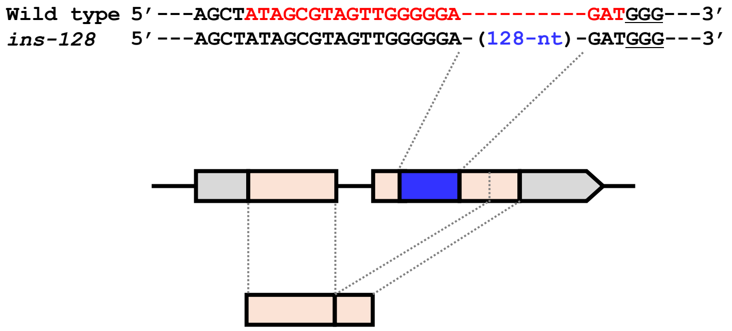

Predicted coding sequence of *Gly m 4-L1* mutant allele in *Gly m 4-L1*$^{128\text{-}ins}$

Figure S9. Predicted splicing pattern from the *Gly m 4-L1 mutant* allele in *Gly m 4-L1*$^{128\text{-}ins}$ plants. Exons are represented by boxes and pentagons, with gray regions indicating untranslated sequences. Pentagons mark the transcriptional endpoints. The blue region denotes the 128-nt insertion. Red sequences of the gRNA target regions are shown, with underlined sequences indicating the protospacer adjacent motif (PAM).

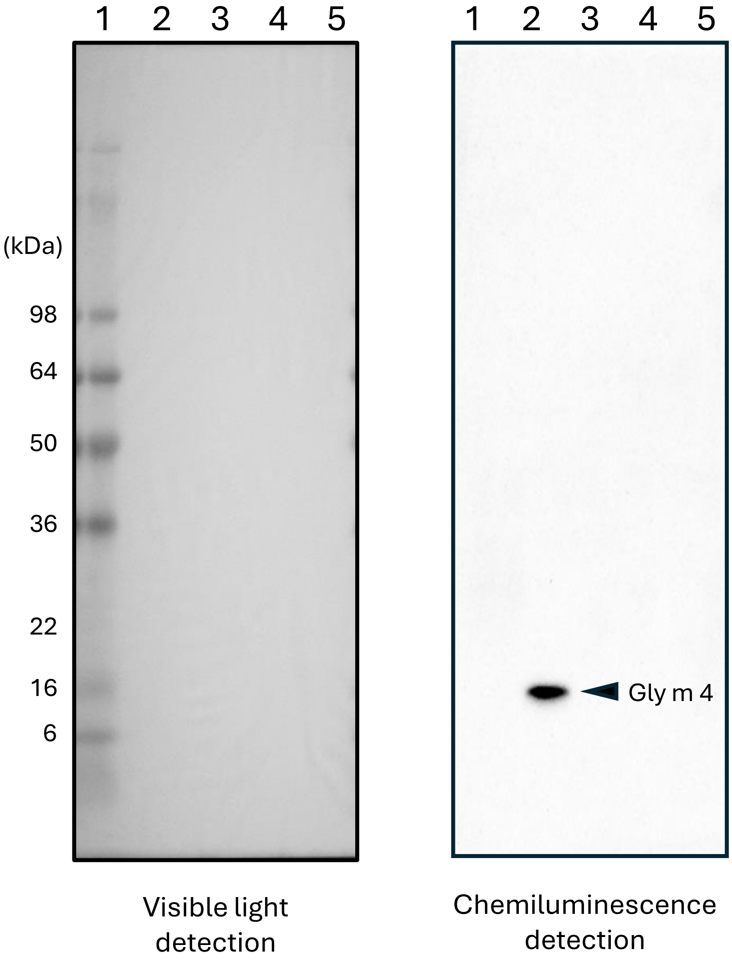

Figure S10. Immunoblot probed with a polyclonal antibody against Gly m 4 protein. Lane 1, protein molecular weigh marker; lane 2, control; lane 3, *Gly m 4-L1*$^{8\text{-}del}$ mutant; lane 4, *Gly m 4-L1*$^{128\text{-}ins}$ mutant; lane 5, *Gly m 4-L1*^{null} mutant. The complete membrane used for immunoblotting is shown to ensure transparency of the experimental procedure. The left panel shows the molecular weight marker signals transferred onto the membrane.

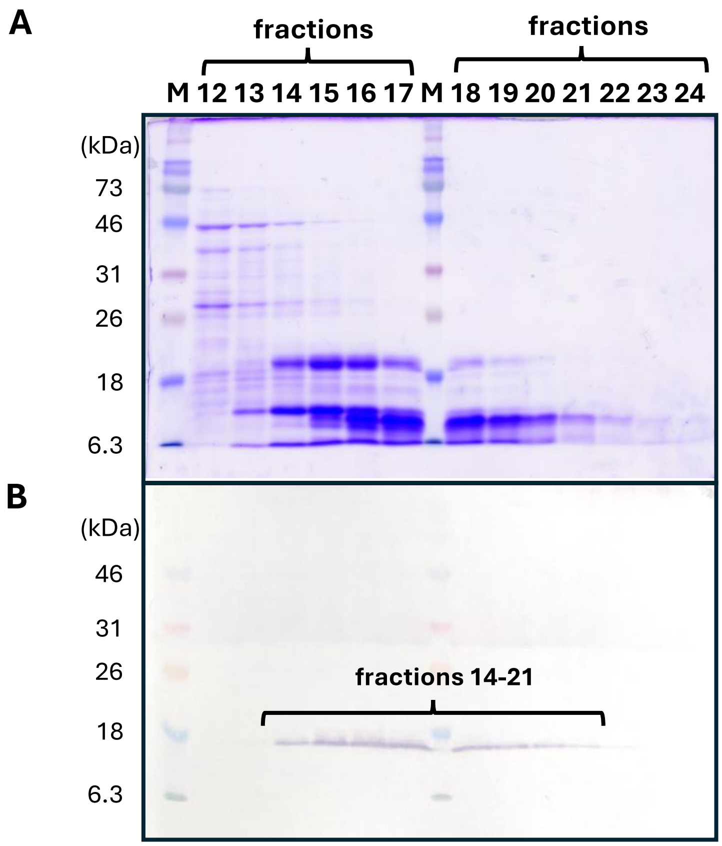

Figure S11. SDS-PAGE and immunoblot analysis of seed proteins from control plants following gel filtration chromatography. **(A)** SDS-PAGE of protein fractions collected from control plants. Proteins were fractionated by gel filtration chromatography using a HiLoad 16/60 Superdex column at a flow rate of 0.7 mL/min, with fractions collected every 3 minutes. Effluents from fractions 12 to 24 were subjected to SDS-PAGE. **(B)** Immunoblot analysis of SDS-PAGE-separated proteins using a polyclonal antibody against the Gly m 4 protein.

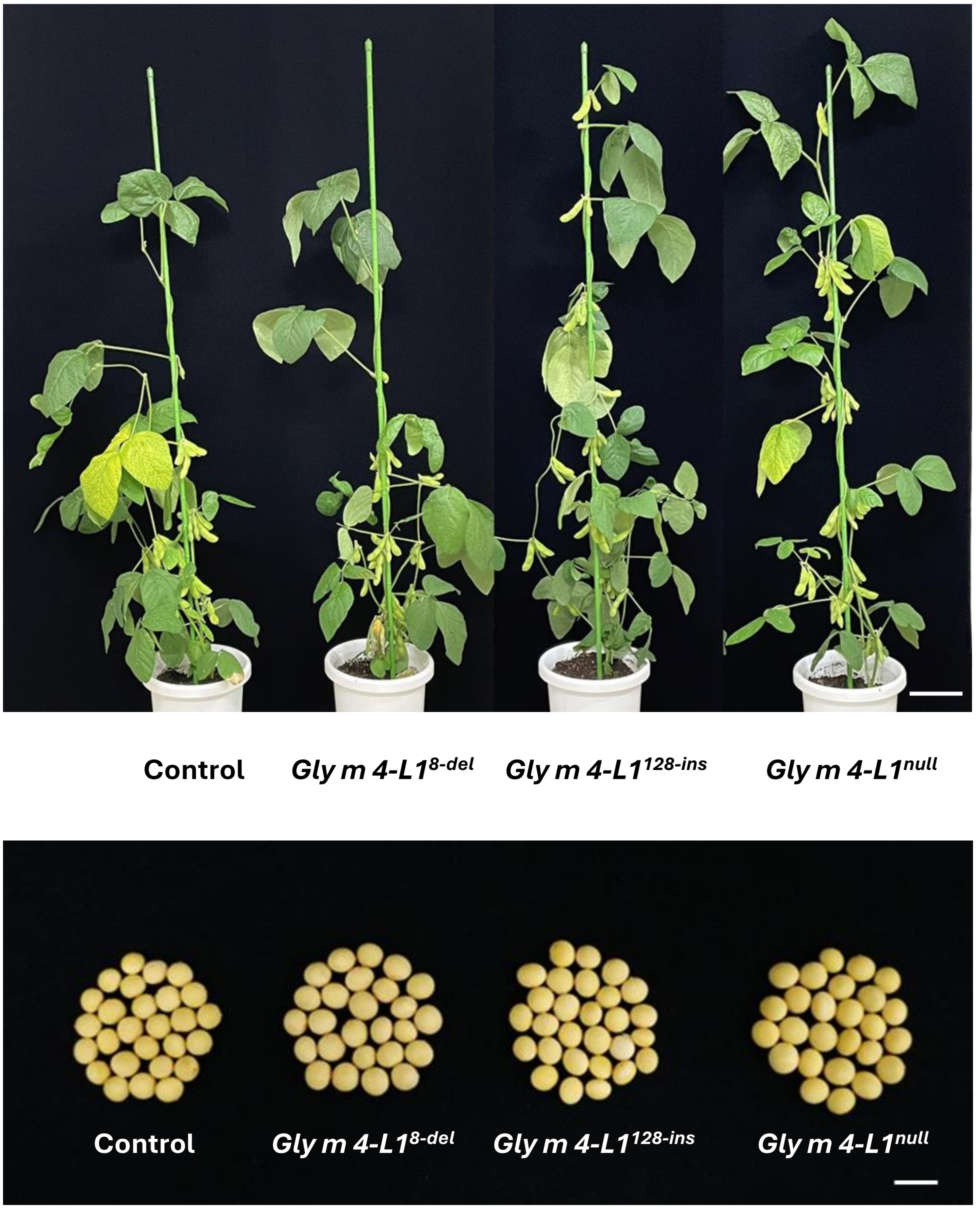

Figure S12. Morphological characteristics of control, *Gly m 4-L1*$^{8\text{-}del}$, *Gly m 4-L1*$^{128\text{-}ins}$, and *Gly m 4-L1*^{null} mutants. Upper panel: whole plant bodies. Scale bar, 10 cm. Lower panel: mature seeds. Scale bar,1 cm.

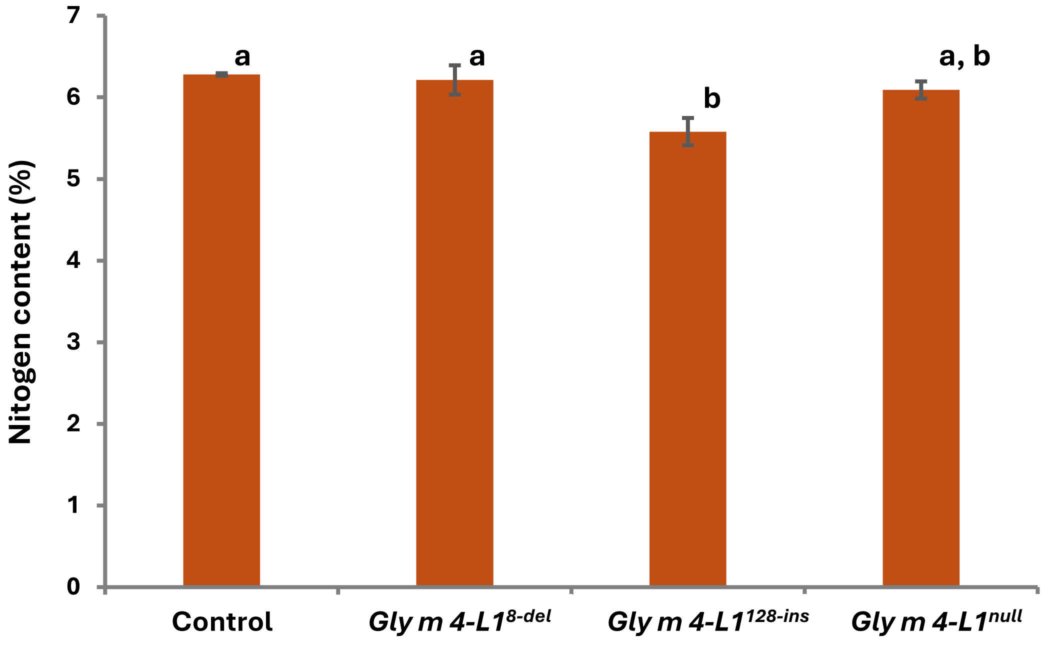

Figure S13. Nitrogen contents in mature seeds of control and *Gly m 4-L1* mutants. Data are means ± SE from three independent trials. Statistically significant differences among the control and mutant plants are represented by different lowercase letters above the bars, based on ANOVA followed by Tukey's HSD test ($p < 0.05$).

Glyma.07G243600.2 (Gly m 4- L1)
Protein sequence coverage: 97%

```
  1 MGVFTFEDET TSPVAPATLY KALVTDADNV IPKAVDAFRS VENVEGNGGP
 51 GTIKKITFLE DGETKFVLHK IEAIDEANLG YSYSVVGGDG LPDTVEKITF
101 ECKLAAGANG GSAGKLTVKY QTKGDAQPNQ DDLKIGKAKS DALFKAVEAY
151 LLAHPDYN
```

Glyma.17G030200.1
Protein sequence coverage: 97%

```
  1 MGIFTFEDET TSPVAPATLY KALVTDADNV IPKAVEAFRS VENLEGNGGP
 51 GTIKKITFVE DGESKFVLHK IESVDEANLG YSYSVVGGVG LPDTVEKITF
101 ECKLAAGANG GSAGKLTVKY QTKGDAQPNP DDLKIGKVKS DALFKAVEAY
151 LLANPHYN
```

Glyma.17G030300.1
Protein sequence coverage: 97%

```
  1 MGVFTFEDET TSPVAPATLY KALVTDADNV IPKAVDAFRS VENLEGNGGP
 51 GTIKKITFVE DGESKFVLHK IESVDEANLG YSYSVVGGVG LPDTVEKITF
101 ECKLAAGANG GSAGKLTVKY QTKGDAQPNP DDLKIGKVKS DALFKAVEAY
151 LLANPHYN
```

Figure S14. Predicted peptide sequences from LC–MS/MS analysis of gel bands corresponding to Gly m 4-sized proteins.
The gel slices were incubated at 37°C with sequencing grade-modified trypsin (Promega) in 50 mM NH_4HCO_3 for 12 h, and the extracted peptides were subjected to liquid chromatography-tandem mass spectrometry (LC-MS/MS) on nano-Advance (AMR) and Q Exactive Plus (Thermo Fisher Scientific). Ion spectrum data were analyzed against UniprotKB with the Mascot Server 2.7.0 (Matrix Science).
